# A site of vulnerability at V3 crown defined by HIV-1 bNAb M4008_N1

**DOI:** 10.1038/s41467-021-26846-z

**Published:** 2021-11-09

**Authors:** Kun-Wei Chan, Christina C. Luo, Hong Lu, Xueling Wu, Xiang-Peng Kong

**Affiliations:** 1 Department of Biochemistry and Molecular Pharmacology, NYU Grossman School of Medicine, New York, NY 10016 USA; 2 Aaron Diamond AIDS Research Center, Columbia University Vagelos College of Physicians and Surgeons, New York, NY 10032 USA

**Keywords:** Adaptive immunity, Cryoelectron microscopy, Structural biology

## Abstract

Identification of vulnerable sites defined by broadly neutralizing antibodies (bNAbs) on HIV-1 envelope (Env) is crucial for vaccine design, and we present here a vulnerable site defined by bNAb M4008_N1, which neutralizes about 40% of a tier-2 virus panel. A 3.2 Å resolution cryo-EM structure of M4008_N1 in complex with BG505 DS-SOSIP reveals a large, shallow protein epitope surface centered at the V3 crown of gp120 and surrounded by key glycans. M4008_N1 interacts with gp120 primarily through its hammerhead CDR H3 to form a β-sheet interaction with the V3 crown hairpin. This makes M4008_N1 compatible with the closed conformation of the prefusion Env trimer, and thus distinct from other known V3 crown mAbs. This mode of bNAb approaching the immunogenic V3 crown in the native Env trimer suggests a strategy for immunogen design targeting this site of vulnerability.

## Abstract (web summary)

Mapping of the HIV Env surface epitopes targeted by broadly neutralizing antibodies (bNAbs) is of great interest for HIV-1 vaccine design. Here, the authors present the 3.2 Å cryo-EM structure of the bNAb M4008_N1 in complex with BG505 DS-SOSIP, an engineered native-like Env trimer and observe that the bNAb epitope is centered at the V3 crown and that M4008_N1 uses its CDR H3 to form an extended β-sheet with the β-hairpin of the V3 crown in a conformation stabilized in the prefusion trimer.

## Introduction

It is generally agreed that identifying the epitopes targeted by HIV-1 broadly neutralizing antibodies (bNAbs) provides crucial guidance for the design of a protective HIV-1 vaccine^1–3^. However, the target for bNAbs on the viral surface, the envelope (Env) glycoprotein, which assembles into a trimeric spike of gp120/gp41 heterodimers, has evolved several escape mechanisms to evade the humoral immune response, including the low spike density on the viral surface^4^, the unstable structure of the trimer, high sequence variation across strains, and masking of epitopes with glycans^5,6^. As a result, bNAbs only emerge several years after infection in a subset of chronically HIV-1 infected individuals^7^.

Advances in the last decade in single B-cell sorting technology, either using direct single memory B-cell screening for neutralization activity^8–10^ or with antigen-specific probes^11^, have led to the identification of many new bNAbs. With the recent success of constructing recombinant native-like Env trimers such as SOSIP^12^ and NFL^13^, even more bNAbs have been identified and characterized^14–17^, and thus antigenic profiles and structural features associated with the native trimer can be interpreted. The epitopes of these bNAbs have been classified into seven conserved regions, which include the first and the second variable region (V1V2)^18–20^, the V3 base glycan supersite^8^, the CD4 binding site (CD4bs)^11,21^, the silent face center^22,23^, the gp120/gp41 interface^15,16^, the fusion peptide (FP)^24^, and the membrane-proximal external region (MPER)^25,26^.

BNAbs M4008_N1 and M1214_N1 were identified recently using a vesicular stomatitis virus (VSV)-based probe that displays membrane-embedded HIV-1 Env trimers^27^. M4008_N1 can neutralize about 40% of a tier-2 panel of 120 primary isolates and, similar to that of M1214_N1, its lineage class-switched to both IgG and IgA. We previously showed that the M1214_N1 epitope is located between V2 and V5 and next to the well-studied CD4bs^27^. However, the epitope of M4008_N1 was unknown. Our data from competition ELISA assays showed that M4008_N1 competes with mAbs targeting the crown region of the V3 loop of gp120, a region known to be immunogenic^28^, but its neutralization does not rely on glycans. Thus, M4008_N1 is distinct from previously known V3 crown mAbs as well as those targeting the V3 base glycan supersite^27^.

To map the epitope of M4008_N1, we determined a cryo-EM structure of the antigen-binding fragment (Fab) of M4008_N1 in complex with BG505 DS-SOSIP trimer at a resolution of 3.2 Å. Our structure reveals that the epitope of M4008_N1 is centered at the V3 crown, in which M4008_N1 uses its CDR H3 to form an extended β-sheet with the β-hairpin of the V3 crown in a conformation stabilized in the prefusion trimer, a binding mode different from that of previously known V3 crown antibodies. Our structural results show how a bNAb can target the crown region of the V3 loop on the prefusion closed Env trimer, and the mode of recognition by M4008_N1 suggests a strategy to target this site of vulnerability in HIV vaccine design.

## Results

### Cryo-EM structure of M4008_N1 Fab in complex with BG505 DS-SOSIP

**Fig. 1:**
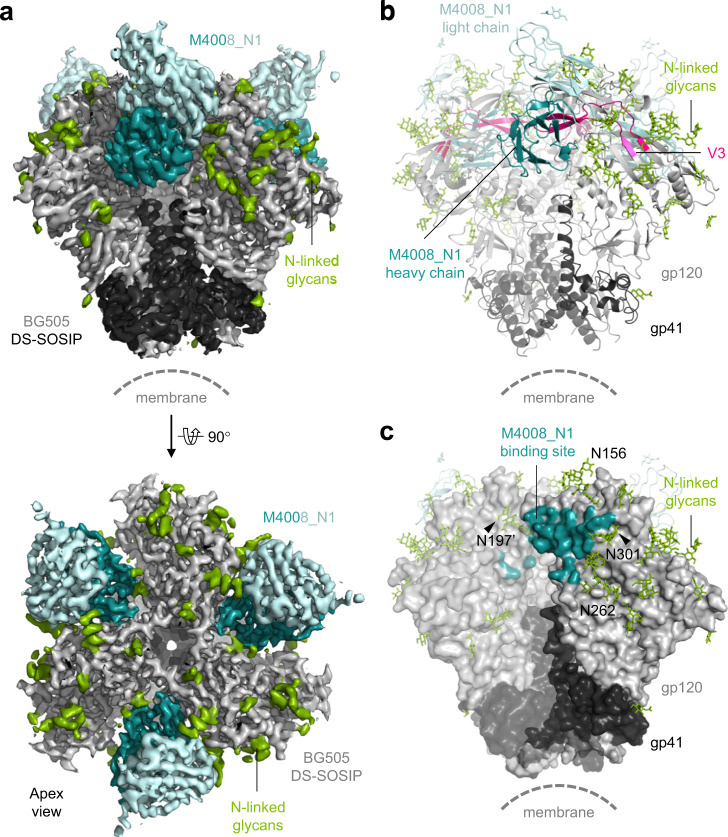
Cryo-EM structure of M4008_N1/DS-SOSIP trimer complex. **a** Cryo-EM 3D reconstruction map of M4008_N1 Fab in complex with BG505 DS-SOSIP in side (upper panel) and apex (lower panel) views. The densities corresponding to the heavy and light chains of M4008_N1 Fab are colored dark and light cyan, respectively, while those corresponding to gp120 and gp41 are colored gray and black, respectively. The observed N-linked glycans on the trimer are colored green. **b** Ribbon representation of the final structural model of the complex. The color usage is the same as in **a** except that the V3 loop is highlighted in pink. The saccharide residues included in the final model are displayed as green sticks for the glycans on gp120/gp41 and as light cyan sticks for the glycan on the light chain of M4008_N1. The constant domain of M4008_N1 Fab was not included in the final structure due to weak densities. **c** The contact surface area of M4008_N1 on the Env trimer. For clarity, the model of M4008_N1 on the primary gp120 is removed, and different levels of surface transparency are used on adjacent gp120/gp41 protomers. For illustration purposes, atoms within 5.0 Å from M4008_N1 are included and colored dark cyan. Key contact glycans around the M4008_N1 binding site on the primary gp120 are labeled.

To map the epitope of M4008_N1 on HIV-1 Env we complexed M4008_N1 Fab with a conformationally stabilized Env trimer, DS-SOSIP.664^29^, derived from the clade A strain BG505, which is one of the trimeric constructs mimicking the native functional trimer^12^. We determined a 3.2 Å cryo-EM structure with a 3:1 Fab:Env trimer stoichiometry (Fig. 1a, b and Supplementary Table 1). Although partially occupied Env trimers (2:1 Fab:Env trimer) were observed in our cryo-EM class averages (Supplementary Fig. 1), there is little difference in the relative orientations among the three gp120 protomers between the fully occupied trimer and the partially occupied one (Supplementary Fig. 2), indicating that the three binding sites on the trimer are equivalent for M4008_N1 binding. Additionally, based on visual inspection, the reconstruction map of the fully occupied trimer has a 3-fold axis. Therefore, C3 symmetry was imposed in the final refinement.

The M4008_N1/DS-SOSIP complex structure showed that while each Fab of M4008_N1 is tightly packed into the crevice formed by two adjacent gp120 protomers (Fig. 1b), it engages primarily one gp120 (primary gp120) on one side of the crevice, with a total interface area of 1357.6 Å^2^, mostly involving the protein component, but also some contacts with surrounding glycans (Fig. 1c). M4008_N1 also engages the neighboring gp120 on the other side of the crevice, with an interface area of 394.1 Å^2^, involving some protein component and a critical glycan (see below).

M4008_N1-bound DS-SOSIP trimer remained in a similar conformation to previously determined prefusion conformation SOSIP trimers, as the binding of M4008_N1 did not alter the arrangement between gp120 protomers or the structure at the gp120/gp41 interface (Supplementary Fig. 3). For example, it aligns well with the ligand-free trimer (PDB ID 4ZMJ) with a Cα RMSD of 0.94 Å, calculated by 1518 aligned atoms (88% of Cα), suggesting that the epitope of M4008_N1 is present on the Env surface in its prefusion state. We further investigated the binding compatibility of M4008_N1 with VRC01 and M1214_N1 by EM visualization of negatively stained ternary complexes of M4008_N1 with one of these two bNAbs on JR-FL gp120 (Supplementary Fig. 4). Our data showed that M4008_N1 binding on monomeric gp120 is compatible with the binding of CD4bs antibody VRC01 and V2V5 corridor antibody M1214_N1. Thus, M4008_N1 binding likely does not alter the conformations of other bNAb epitope regions on the trimer.

### A large epitope surface at V3 crown surrounded by key glycans

**Fig. 2:**
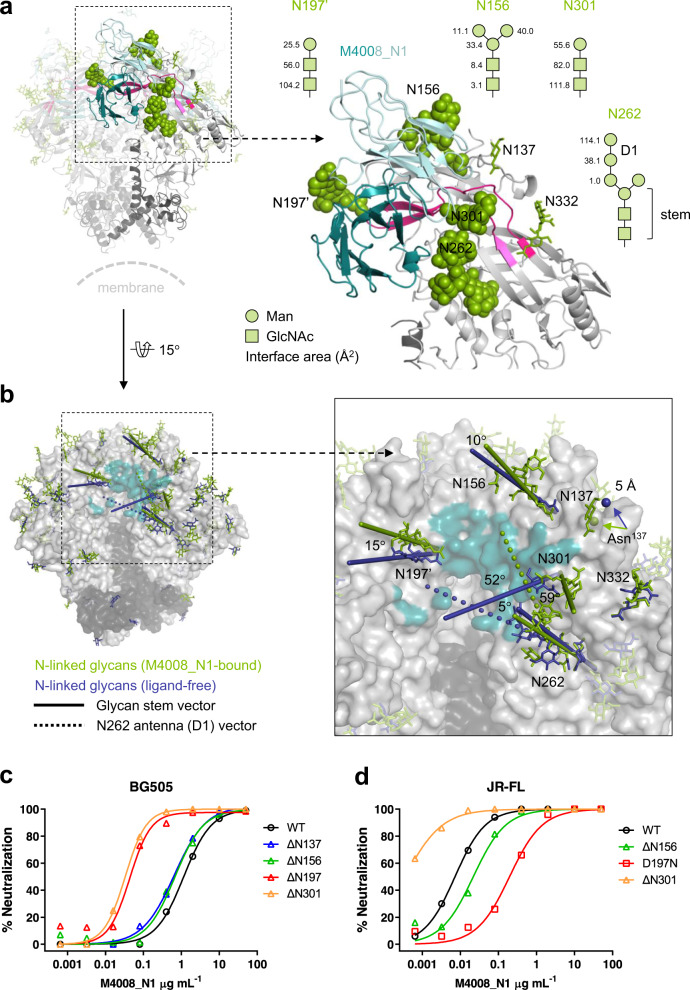
N-linked glycans at the M4008_N1 binding site. **a** Contact glycans at the M4008_N1 binding site. The surrounding glycans involved in the binding sites of M4008_N1 on the primary gp120 of the trimer are displayed as green spheres. The interface area (Å^2^) of each saccharide residue is listed next to the corresponding schematic drawings. **b** Glycan orientation changes upon M4008_N1 binding. A structural alignment of the M4008_N1-bound SOSIP trimer onto the ligand-free SOSIP trimer (PDB ID 4ZMJ) shows the orientation change of each surrounding glycan upon M4008_N1 binding. The orientation vector corresponding to each glycan was defined by the direction from Cα of the attached Asn to C4 of the first GlcNAc, while the vector for the D1 arm of glycan N262 was calculated from C1 of the first Man (M1) to C1 of the second Man (M2). Due to a lack of glycan at Asn^137^ in 4ZMJ, the conformational change upon M4008_N1 binding at position 137 is displayed with corresponding Cα atoms. **c**, **d** Glycan influence on M4008_N1 neutralization. Neutralization tests were carried out for M4008_N1 against glycan-modified Env variants for strains BG505 (**c**) and JR-FL (**d**). Note that in the case of BG505, removal of glycans next to the M4008_N1 binding site improved its neutralization potency, especially for glycans of N301 of the primary gp120 and N197 of the neighboring gp120, while in the case of JR-FL, introducing the glycan of N197 of the neighboring gp120 reduced its potency. Neutralization was performed in duplicate wells, and the data are represented as means.

The epitope of M4008_N1 occupies a large protein surface area, with a diameter of about 30 Å on the surface of the primary gp120, surrounded by a number of N-linked glycans (Fig. 1c). In our M4008_N1/DS-SOSIP structure, 18 of the 28 potential N-linked glycosylation sites on each gp120/gp41 heterodimer were observed with densities, allowing modeling of saccharide units into them (Fig. 2 and Supplementary Fig. 5). The epitope of M4008_N1 is bordered on the top, if we view the Env trimer from its side with the trimer apex pointing up, by glycan N156 (hereafter each glycan is referred to by the linked Asn [N] residue number), on the left by glycan N197’ from the neighboring gp120, and on the right by glycan N301 (Fig. 2a). In addition, the C2 glycan N262 reaches up to fortify the right border with glycan N301. M4008_N1 forms direct contacts with these four glycans with a total interface area of 684.3 Å^2^, about 39% of the overall interface area between M4008_N1 and the Env trimer. Interestingly, these glycans are among the most conserved glycans of Env^30^. The glycan contacts are mostly at the stalk region, except oligomannose-type glycan N262, which contacts M4008_N1 through the terminus of its D1 arm.

The large protein surface contact area of M4008_N1 on the Env trimer implies the shallowness of this glycan-enclosed region. This shallowness can be evaluated in general by calculating the ratio of the interface area between antibody and Env protein surface to that between antibody and Env glycan (Supplementary Fig. 6 and Supplementary Data 1), in which a higher ratio value reflects a broader openness of the binding region. The ratio of the epitope region for M4008_N1 is 1.5, which is comparable with that of the V2V5 corridor bNAb M1214_N1 (1.7); this ratio is smaller than the mean ratio for CD4bs bNAbs (2.6), but is larger than the mean ratios for other vulnerable sites, including the V3 base region (1.0), the gp120/gp41 interface (0.9), the fusion peptide region (0.9), the V1V2 apex (0.8), and the silent face (0.3). Thus, while less shallow than the CD4bs region, the M4008_N1 epitope is shallower than the epitopes of most other vulnerable sites. A shallow epitope region requires a relatively less protruding CDR H3 loop for recognition, as in the case of M4008_N1 (see below).

By comparing the orientations of glycans from our structure of M4008_N1-bound DS-SOSIP with that of the unliganded SOSIP trimer (PDB ID 4ZMJ), in which the glycans of interest were not disturbed by any antibodies (Fig. 2b), we observed a range of orientation changes of the M4008_N1 contact glycans, likely caused by M4008_N1 binding. Most prominently, glycan N301 exhibited a large movement in which it tilted away from the center of the binding site by an angle of 59°. The changes of glycans N156 and N197 were subtle, with tilted angles of only 10° and 15°, respectively. Unlike these three glycans with their glycan bases bordering the M4008_N1 binding site, glycan N262, located at the gp120 C2 region, extends its oligosaccharide branches towards the M4008_N1 binding site, into a groove between the V1V2 stem of the gp120 inner domain and the C4 region of the outer domain. Binding of M4008_N1 caused only a slight movement at its GlcNAc_2_ stem but a 52° shift of its D1 arm.

We created a panel of pseudovirus variants with altered Env glycans around the M4008_N1 binding site and assessed their neutralization by M4008_N1 (Fig. 2c, d and Supplementary Table 2). The removal of glycan N197 or N301 from strain BG505 substantially increased its neutralization sensitivity to M4008_N1 (Fig. 2c), but removal of glycan N156 led to only a slight increase of its neutralization sensitivity, consistent with their areas of contacts against M4008_N1 (Fig. 2a) and the scales of the movements upon M4008_N1 binding (Fig. 2b). The prominence of glycans N197 and N301 on the neutralization of M4008_N1 can also be seen from the JR-FL variants of these glycans (Fig. 2d). Firstly, the WT of JR-FL, which naturally lacks glycan N197, is over 100-fold more sensitive to M4008_N1 neutralization than the WT of BG505 (Supplementary Table 2). The addition of glycan N197 for strain JR-FL resulted in a 33-fold drop of neutralization sensitivity to M4008_N1, suggesting the protective role of glycan N197 by its shielding of the V3 crown region of the neighboring gp120. Secondly, the removal of glycan N301 in JR-FL increased its neutralization sensitivity over 30 fold. These results are comparable with previous binding analyses using variants designed based on monomeric gp120^27^, further supporting the conclusion that the neutralization of M4008_N1 is sensitive to the surrounding glycans but does not rely on them.

We also investigated the next layer of glycans encircling the M4008_N1 binding site, including glycans N137 and N332. Glycan N137 is on the V1 loop, located between glycans N156 and N301. A structural comparison revealed a conformational change of the V1 loop upon M4008_N1 binding by a slight rotation, resulting in a ~5 Å shift around the M4008_N1 binding site (Fig. 2b). The removal of glycan N137 from strain BG505 slightly increased its neutralization sensitivity to M4008_N1 (Fig. 2c and Supplementary Table 2), and the removal of glycan N332 also increased its neutralization sensitivity to M4008_N1 as we previously reported^27^; the removal of these glycans may result in more space for glycan N301 to adapt to M4008_N1 binding.

### β-sheet interaction of CDR H3 of M4008_N1 and the V3 crown

**Fig. 3:**
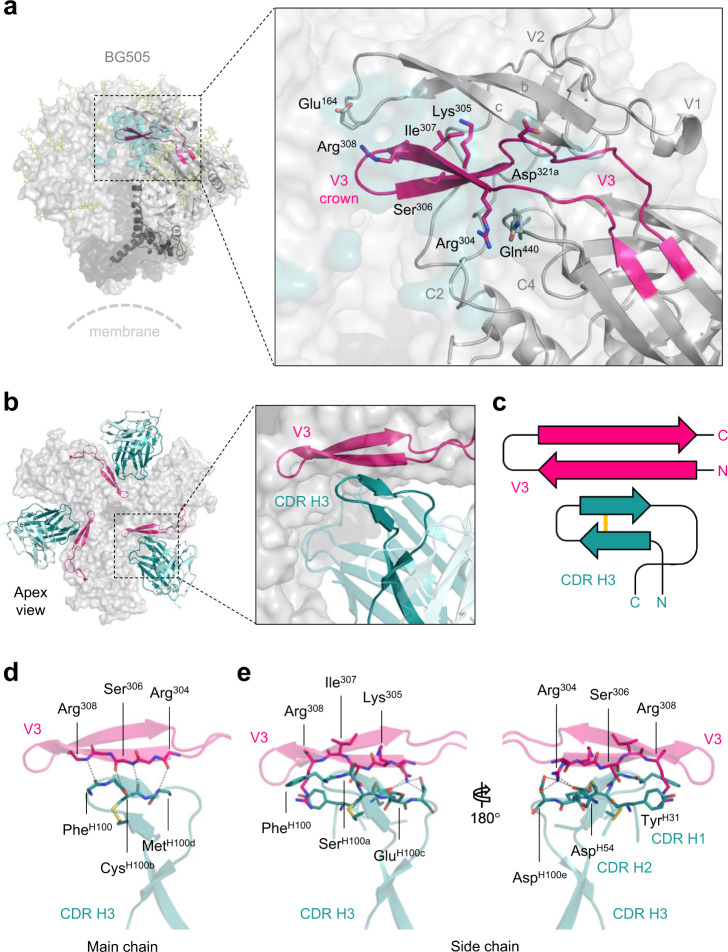
β-sheet interaction of CDR H3 of M4008_N1 and the V3 crown. **a** The epitope of M4008_N1. Protein components that comprise the M4008_N1 binding site on gp120 are labeled, and selected residues involved in M4008_N1 contact are displayed in sticks. **b** β-sheet interaction of CDR H3 and V3 crown. The binding of M4008_N1 to gp120 resulted in the formation of an extended β-sheet consisting of CDR H3 hammerhead and the V3 crown hairpin. **c** Schematic of the four stranded β-sheet formed by the CDR H3 of M4008_N1 and the crown of V3. Note that the CDR H3 of M4008_N1 has an intra-loop disulfide bond (yellow). **d**, **e** Detailed interactions between epitope/paratope main-chains (**d**) and their side chains (**e**). The side chains of residues involved in the interactions are shown as sticks.

M4008_N1 mainly uses its heavy chain to interact with the protein component of gp120 primarily at the V3, with additional contacts at the turn between strands b and c of V1V2, as well as C2 and C4 of gp120 (Fig. 3a and Supplementary Table 3). These regions form a flat surface in the crevice between two adjacent gp120 protomers and likely exist only in the prefusion closed form of the Env trimer, which requires the V3 crown to be tightly packed under the V1V2 barrel and partially shielded by a couple of glycans.

The predominant interaction between M4008_N1 and the V3 is established through the CDR H3 loop and the N-terminal strand of the V3 crown (Fig. 3b, c). The CDR H3 loop of M4008_N1 consists of 17 residues (Kabat definition^31^) derived mostly from V-D-J junctions, with an intra-loop disulfide bond between Cys^H97^ and Cys^H100b^, and it forms the shape of a hammerhead like that of PG9/PG16, though not as prominently protruding from the body of the antibody^18,32–34^. This hammerhead engages the V3 crown hairpin using its disulfide bond-stabilized β-hairpin by forming an intermolecular antiparallel 4-stranded β-sheet (Fig. 3c), so that the strand at the top of the hammerhead aligns with the N-terminal strand of the V3 crown, with backbone hydrogen bonding between residues Phe^H100^, Cys^H100b^, and Met^H100d^ from CDR H3 of M4008_N1 and corresponding residues Arg^308^, Ser^306^, and Arg^304^ at the N-terminus of the V3 crown (Fig. 3d). As we previously described, the V3 crown hairpin can have two conformations, distinguished by the location of a hydrophobic core formed by the highly conserved Ile^307^ and Ile^309^ on the N-terminal strand and a hydrophobic residue on the C-terminal strand^35^. In the prefusion closed form trimers, the V3 crown has the conformation also typified by that bound by V3 crown mAbs such as 2557 or 1334^36,37^ (Supplementary Fig. 7). However, the extended β-sheet binding mode allows M4008_N1 access to the V3 crown in the context of the prefusion closed form trimer, unlike other V3 crown mAbs that do not bind the closed form trimer.

In addition to the main chain interaction, side chains of several residues in the CDR H3 of M4008_N1 are involved in interactions with the conserved structural elements in the V3 crown, focusing on the polar side chains of the V3 crown hairpin (Fig. 3e). At the N-terminal end of V3 crown, the relatively conserved positively charged Arg^304^ can form salt bridges with Asp^H100e^ of the CDR H3 hammerhead on one side and Asp^H54^ of CDR H2 on the other side. At the other end of V3 crown, Arg^308^ is stacked between the side chains of Phe^H100^ of CDR H3 and Tyr^H31^ of CDR H1.

**Fig. 4:**
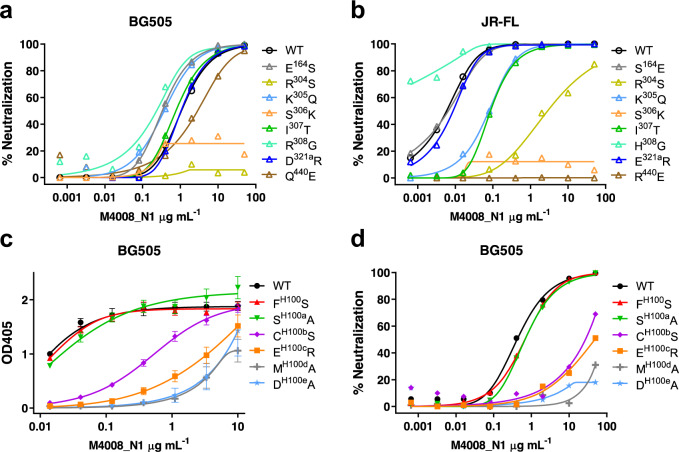
Assessment of key residues at the interface between M4008_N1 and the Env trimer. **a**, **b** Neutralization analyses of M4008_N1 against various mutated Env variants from strain BG505 (**a**) and JR-FL (**b**). **c**, **d** ELISA (**c**) and neutralization analyses (**d**) of M4008_N1 CDR H3 variants against BG505. Neutralization was performed in duplicate wells, and the data are represented as means. ELISA was performed in duplicate wells for three independent experiments, and the data shown are means ± SEM.

We created a panel of variants of BG505 and JR-FL with key residues at the epitope site mutated (Fig. 4a, b and Supplementary Table 4). Our data showed that most of these residue substitutions did not drastically alter their neutralization sensitivity to M4008_N1, as the main chain interaction is the signature of the β-sheet interaction between CDR H3 and the V3 crown. However, three variants of individual mutations at positions 304, 306, and 440, i.e., Arg^304^Ser (R^304^S), S^306^K, and R^440^E (for JR-FL), in fact knocked out the neutralization activity of M4008_N1. As described above, residue Arg^304^ is an important residue that contributes to the antibody-antigen interaction by forming two salt bridges (Fig. 3e); thus, the disruption of these salt bridges through the R^304^S mutation is likely the reason for M4008_N1’s drastically reduced neutralization activity. Similarly, Ser^306^ is another key residue located at the center of the buried surface between M4008_N1 and its epitope, and thus introducing a charged residue there abolished the neutralization activity of M4008_N1. In the case of residue 440 (whose side chain is next to the negatively charged Asp^H100e^) for JR-FL, mutation of R^440^E drastically altered the surface charge (Supplementary Fig. 8), thus it knocked out the neutralization of M4008_N1, while Q^440^E for BG505 resulted in a smaller change in surface charge, thus this mutation only slightly reduced its sensitivity of neutralization.

We also tested the effect of key CDR H3 residues that interact with the N-terminal strand of the V3 crown. Sequence substitution of these residues, particularly at positions H100b, H100c, H100d, and H100e substantially reduced M4008_N1’s binding and neutralization activities to BG505 (Fig. 4c, d and Supplementary Table 5). As the intra-loop disulfide bond plays an important role to stabilize the hammerhead-shaped conformation of CDR H3 for binding, mutation of Cys^H100b^ significantly decreased its binding and neutralization activities to the trimer.

Our structure can also help to explain the neutralization breadth of M4008_N1, as we showed previously that 45 of 120 tested strains were neutralized (IC_50_ ≤ 50 μg mL^−1^) by M4008_N1^27^. As Env glycans are not critical for M4008_N1 recognition, protein sequence variation of the epitope region is likely the primary determinant for the neutralization breadth of M4008_N1. A sequence analysis of all 120 strains indicated that Arg^304^ (95% identical), Lys^305^ (73% identical) at the N-terminal strand of V3, and residue 316 (83% is Ala^316^ or Thr^316^) at the C-terminal strand are relatively important for neutralization. Indeed, of the 75 strains resistant to M4008_N1 neutralization, 32 (43%) mutated to non-Arg^304^ and/or Lys^305^, and 21 (28%) mutated to non-Ala^316^ or Thr^316^ (Supplementary Fig. 9 and Supplementary Data 1). In addition, a negatively charged Glu residue at position 440 is also associated with M4008_N1 neutralization resistance, which is likely not compatible with CDR H3 residue Asp^H100e^ for the resistant strains. Of the 34 resistant strains that contain all three residues identified above as important for neutralization (Arg^304^, Lys^305^, and Ala^316^ or Thr^316^), 18 strains harbor a Glu^440^. In total, 41 neutralized strains harbor this three-residue combination, while 59 resistant strains either lack this combination or harbor Glu^440^; thus, these residue variations can provide an explanation for about 83% (100/120) of the neutralization breadth. Furthermore, the insertion of an N-linked glycosylation site at position 442, which is located between glycans N262, N301 and N332 (Supplementary Fig. 10), may also contribute to M4008_N1 resistance, as only 16% (7/45) of neutralized strains contain both N332 and N442, while 32% (24/75) of non-neutralized strains have both glycans (Supplementary Data 1). Taken together, these can explain the majority of the neutralization breadth of M4008_N1.

### Structural basis of affinity maturation of M4008_N1

**Fig. 5:**
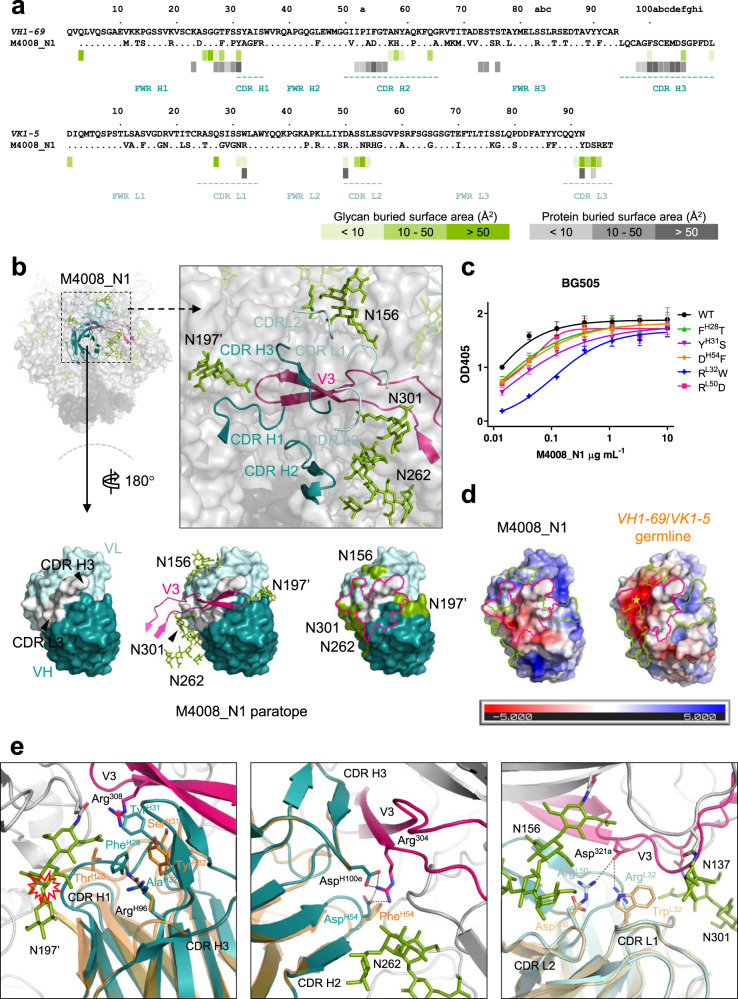
Somatic hypermutations in the VH/VL regions of M4008_N1. **a** Sequence alignment of *V* gene-encoded regions of heavy and light chains of M4008_N1 and their germline sequences. The residues involved in protein and glycan contacts (defined as the paratope residues) are indicated underneath with gray and green bars, respectively, and each bar’s color intensity is proportional to the residue’s buried surface area. **b** The paratope of M4008_N1. Upper panel: the paratope in the context of the epitope region. All of the CDR loops of M4008_N1 are involved in epitope contact and are shown as ribbons. Lower panel: the paratope surface of M4008_N1. Depicted from left to right are the locations of the VH/VL regions, the V3 loop in the context of the paratope, and the outline of the paratope. The regions that involve glycan or V3 contacts are highlighted with green patches and a pink line, respectively. **c** ELISA analysis of different single residue germline-reverted M4008_N1 variants. Analysis was performed in duplicate wells for three independent experiments, and the data shown are means ± SEM. **d** Differences of the electrostatic potential surfaces of M4008_N1 and its *VH1-69*/*VK1-5* germline precursor, with the red indicating negative and blue positive potentials (in units of *k*_B_*T*/*e*_c_). The germline precursor surface was calculated from its structural model. Note the negative charge area around the precursor residue Asp^L50^ indicated by a yellow star. **e** Superimposition of M4008_N1 (colored in cyan) and the *VH1-69*/*VK1-5* germline precursor model (colored in orange). Note that the residues of the germline precursor would create clashes with glycan N197’ (left panel), lose a network of salt bridges with the side chain of Arg^304^ (middle panel), and lose complementary charge interactions with Asp^321a^ (right panel).

HIV-1 bNAbs have been characterized by high levels of somatic hypermutation (SHM)^38^. M4008_N1, derived from *VH1-69* and *VK1-5* germline genes, contains 34% and 33% somatically mutated residues within the *V* gene-encoded regions of the heavy and light chains (VH/VL), respectively (Fig. 5a and Supplementary Table 6). An analysis of the M4008_N1 paratope revealed that 63% of the paratope surface distributes on the VH/VL regions, involving all of the CDR loops as well as frame-work region (FWR) H1, FWR H3, and the N terminus of both heavy and light chains, in which 14 paratope residues were altered through SHM (Fig. 5a, b).

To elucidate the correlation between SHM in M4008_N1 VH/VL regions and the binding affinity of M4008_N1 with Env, we investigated five of these mutated residues successively by changing them back to the germline-encoded residues, and assessed the binding activities of these reverted-to-germline variants with BG505 DS-SOSIP trimer (Fig. 5c and Supplementary Table 7). Note that these reverted-to-germline mutations reduced but did not completely eliminate the binding affinity of M4008_N1 to its antigen.

To gain a structural understanding of the affinity maturation of M4008_N1, we created a model for the *VH1-69*/*VK1-5* germline precursor antibody using published structures that share identical germline sequences of *VH1-69* and *VK1-5* antibodies (Fig. 5d). Structural comparison of the germline precursor antibody and the ligand-bound matured M4008_N1 revealed that affinity maturation of M4008_N1 was likely driven by three distinct structural mechanisms (Fig. 5e). First, a conformational change in CDR H1 (Fig. 5e; left panel). In the germline precursor, Tyr^H32^ in CDR H1 formed an intramolecular cation-π interaction with the conserved residue Arg^H96^ at the N-terminal end of CDR H3. This cation–π interaction is highly preserved among antibodies with *VH1-69* germline sequence. In contrast, in matured M4008_N1, somatic mutation of Tyr^H32^ to Ala^H32^ disabled this interaction. Instead, the substitution of Phe^H28^ for Thr^H28^ took the place of Tyr^H32^ to form a cation–π interaction with Arg^H96^. This mutation-driven swapping of a cation–π interaction re-organized the conformation of CDR H1 and minimized a structural clash with glycan N197’. Second, VH chain polar interactions (Fig. 5e; left and mid panels). A number of polar interactions with the V3 crown were generated through somatically mutated residues in the VH region, including a Ser to Tyr substitution at position 31 in CDR H1 to create a cation–π interaction with Arg^308^ of V3 and a Phe to Asp substitution at position 54 in CDR H2 to allow the formation of a salt bridge between Asp^H54^ and Arg^304^ of V3. Despite M4008_N1 recognition of V3 occurring primarily through its CDR H3, these additional polar interactions enhanced binding activities of M4008_N1. Third, VL chain charge interactions (Fig. 5e; right panel). The paratope surface against V3 was extended through positively charged residues on the VL region. Residues Trp^L32^ and Asp^L50^ from CDR L1 and CDR L2, respectively, were both somatically mutated to Arg to create a positively charged surface, complementing the nearby V3 stem residue Asp^321*a*^, which is spatially located between glycans N156 and N301 and thus partially shielded until M4008_N1 binding induces the movement of N301.

Altogether, SHM in VH/VL regions enhanced the binding affinity of M4008_N1 toward the Env trimer. This increase might have been achieved through distinct structural mechanisms, including preorganization or rigidification of the CDR loops, improved shape complementarity and electrostatic potential at the interface against glycans, and additional polar interactions with the epitope.

## Discussion

**Fig. 6:**
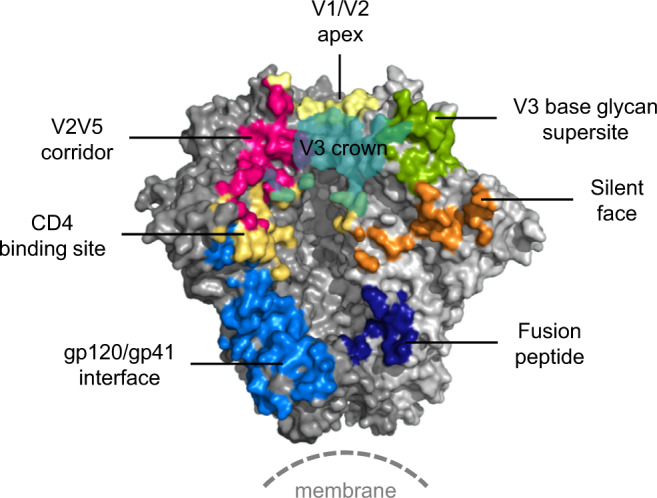
The V3 crown vulnerable site in the context of other sites on an HIV-1 spike. All the vulnerable sites defined by currently identified bNAbs except the MPER region are displayed on the HIV-1 Env trimer.

It is essential for HIV vaccine development to map the epitopes targeted by bNAbs on the surface of HIV Env, and to determine the structural details of their antibody–antigen interactions at the molecular level in order to inform immunogen design^39^. Accordingly, many human HIV bNAbs have been identified and characterized in the last decade and their epitopes carefully mapped on the Env surface^8,40,41^. These bNAb-targeting sites are considered vulnerable sites of HIV-1 and have been found to cover major surface areas on the Env spike. We previously showed that bNAb M1214_N1 targets an elongated region between V2 and V5 next to the well-characterized CD4bs^27^. Here we present a vulnerable site defined by bNAb M4008_N1, which has about 40% neutralization coverage. Our 3.2 Å cryo-EM data revealed the molecular details of its interaction with the Env trimer, primarily via a β-sheet interaction engaging the V3 crown hairpin. The M4008_N1 epitope encompasses an extended surface area centered at the V3 crown and is different from any of the previously known bNAb targeting sites; we therefore designate it as the V3 crown vulnerable site (Fig. 6).

Interestingly, bNAbs M1214_N1 and M4008_N1 were isolated using a VSV-displayed HIV-1 envelope trimer as the bait and were shown to have class-switched to both IgG and IgA^27^. This is both the first demonstration of such class-switching for HIV-1 bNAbs and the first broadly neutralizing IgA HIV-1 antibodies identified to date. Such antibodies may have unique binding sites that are different from those that have class-switched only to IgG in infected patients. This question deserves further investigation.

The M4008_N1 epitope and its interaction with the Env trimer have several salient features. The most notable is the large flat protein surface area of the epitope, which has a diameter of about 30 Å. The Env trimer surface is mostly covered with glycans^30^, and the exposed protein surface areas are usually small or narrow and hard to reach between these glycans by antibodies. In addition, the location of individual glycans can vary between strains or drift in reaction to immune responses; this makes it even harder for antibodies to take a foothold at the exposed protein surface. Thus, a large, exposed surface area like the M4008_N1 binding site is rare. One exception is the CD4bs, which is slightly larger than the V3 crown site, and is also exposed because it has to be available to interact with the CD4 receptor to mediate viral entry. Functionally, the V3 crown is required to interact with the co-receptor CCR5/CXCR4^42^, but this interaction only becomes available after CD4 binding, which triggers a large conformational change of the Env trimer that allows V3 to swing out from its packed conformation below V1V2 in the prefusion state. The requirement for V3 to drastically change conformation may be the reason for the limited number of glycans around the V3 crown region, i.e., the M4008_N1 epitope site.

As mentioned above, the relatively flat protein surface of the V3 crown site requires that the V3 crown be tightly sequestered under V1V2. Thus, M4008_N1 can only engage the V3 crown by a β-sheet interaction; this makes M4008_N1 very different from other known V3 crown antibodies. We and others have previously characterized many of these V3 crown mAbs and classified their binding modes into several classes^35–37,43–45^. However, all of these binding modes require sufficient contact at the tip of the V3 hairpin, making them incompatible with the packed V3 crown in the prefusion closed form Env trimer. This is also likely the reason why those previously known V3 crown mAbs can only neutralize tier-1 viruses, which presumably display open or partially open Env trimers. It is interesting to point out that the tier 1-neutralizing V3 crown mAbs are often used in negative selection during the SOSIP purification process to remove trimers that have the V3 crown exposed^29^.

Another notable characteristic of M4008_N1 is that its neutralization capability is glycan sensitive but not glycan dependent. As we have shown, removing the surrounding glycans of its Env epitope only increases its neutralization potency. In this regard, M4008_N1 is similar to bNAbs targeting the CD4bs, and removing the surrounding glycans allows a better access to the epitope site, thus improving the neutralization potency of the bNAb^46^. This characteristic of M4008_N1 is different from bNAbs such as PG9/PG16 and those targeting the V3 base glycan supersite^18,32,47^. Env glycans that interact with these bNAbs are an integral part of their epitopes and removal of the interacting glycans destroys their neutralization capability. Additionally, these bNAbs often have a long CDR H3 protruding from the body of the antibody that enable it to reach between the glycans to make contacts with the protein surface. The relatively exposed protein surface of the M4008_N1 epitope does not require such a long protruding CDR H3 for protein contacts on the Env surface.

The unique characteristics of the M4008_N1 epitope and its mode of interaction with the V3 crown suggest a strategy to induce M4008_N1-like antibody responses by a V3 priming and heterologous boosting with stabilized Env trimer to target this V3 crown vulnerable site. The V3 crown is the most immunogenic region of gp120, and thus V3 crown-directed antibodies are produced by all HIV-1 infected patients and are relatively straightforward to elicit by vaccines^48–50^. Most of these V3 antibodies are likely similar to those structurally characterized V3 crown mAbs that are not compatible with the closed form Env trimer, but since the V3 crown hairpin is a common structure observed between the epitope of M4008_N1 and that of other V3-directed mAbs, one can anticipate that antibodies that engage the N-terminal strand by a β-sheet interaction, like M4008_N1, are inducible by V3 immunogens. However, the V3 in such immunogens should be stabilized, either by a scaffold or in a context like that of gp120, as the linear crown or cyclic V3 peptides alone are too flexible, illustrated by poor binding activities to M4008_N1 (Supplementary Fig. 11 and Supplementary Table 8). If M4008_N1-like antibody responses can be induced by such V3 immunogens, these responses can then be further boosted with a subsequent immunization using V3-stabilized Env trimers in a heterologous boosting vaccination strategy^51–54^. Early versions of the SOSIP immunogens were known to induce non-neutralizing V3 antibodies, but some V3-stabilized versions seemed to be able to significantly reduce non-neutralizing V3 antibody responses^55^. These V3-stabilized SOSIP trimers can therefore be used for the heterologous boost to induce M4008_N1-like antibody responses.

*VH1-69*, the variable gene encoding M4008_N1, is one of the most commonly used antibody variable genes^56,57^. *VH1-69* has been shown to encode bNAbs against HIV, HCV, RSV, and influenza viruses, etc.^56,58^. This common gene usage is often attributed to the hydrophobic residues in its CDR H2, particularly residues at positions 53 and 54, which can form hydrophobic interactions with the antigen. In the case of M4008_N1, these two residues are mutated in the mature antibody; our reverting Asp to Phe at position 54 only slightly reduced the binding of M4008_N1 to its epitope (Fig. 5c). M4008_N1 CDR H3 also has the specific feature of a disulfide bond-stabilized hairpin that forms the β-sheet interaction with V3 crown in the Env trimer. This disulfide motif in CDR H3 has been estimated to be not uncommon and potentially inducible by vaccination^59^.

The heterologous boosting strategy has been demonstrated to be effective in recent HIV immunogenicity studies^51–54^. In one case it was used to induce neutralizing antibody responses against a FP (fusion peptide) epitope^52^. Animals were first immunized with KLH-conjugated HIV-1 FP immunogens, and subsequently boosted with SOSIP trimers. In another study, the heterologous boosting strategy was used to induce antibody responses targeting the CD4bs^51^. In this case, the animals were first immunized with trimers in which glycans near the CD4bs were removed and then boosted with glycan-restored trimers, and it demonstrated that indeed bNAb responses were induced targeting multiple sites of vulnerability. Taken together, these studies suggest that the heterologous boosting strategy may be useful in inducing bNAb responses targeting the V3 crown vulnerable site.

## Methods

### IgG production and purification

M4008_N1 for cryo-EM was expressed by transient transfection in FreeStyle 293-F cells (Invitrogen). Briefly, IgG plasmid DNA was co-transfected into cells at a 1:1 ratio of heavy:light chain DNA with 25 kDa linear polyethylenimine (PEI; Polysciences) at a 1:3 ratio of DNA:PEI. Cell culture supernatants were harvested after five days, 0.2 μm filtered, and purified on a HiTrap Protein A HP column (GE Healthcare) using an ÄKTA pure protein purification system (GE Healthcare), with an equilibration buffer of 20 mM Tris pH 7.5 with 150 mM NaCl and an elution buffer of 100 mM glycine-HCl pH 2.8. Elution fractions were neutralized with Tris pH 8.8, confirmed on a 12% SDS-PAGE gel, dialyzed to phosphate buffered saline (PBS), and concentrated. M4008_N1 variants for ELISA analyses were generated using the QuikChange II XL Site-Directed Mutagenesis Kit (Agilent). M4008_N1 heavy chain or light chain plasmid DNA was used as the template DNA, and mutagenesis primers were designed with Agilent’s QuikChange Primer Design program and ordered from Sigma (Supplementary Table 10). Variants were confirmed by sequencing (Psomagen), and WT and variants were subsequently expressed by small-scale transient transfection in ExpiCHO-S cells (Thermo Fisher Scientific). Briefly, the plasmid DNA was co-transfected into a 20 mL cell culture at a 1:1 ratio of heavy:light chain DNA with ExpiFectamine CHO reagent following the ExpiCHO Expression System Max Titer Protocol. ExpiCHO Feed and Enhancer were added according to the protocol provided by the vendor, and cell culture supernatants were harvested between day 7–10 and 0.2 μm filtered. Supernatants were then purified on a HiTrap Protein A HP column (GE Healthcare) with an equilibration buffer of 25 mM Tris pH 7.5 with 25 mM NaCl and an elution buffer of 100 mM citrate pH 3 with 87.5 mM NaCl. Elution fractions were neutralized, confirmed on a 12% SDS-PAGE gel, dialyzed to PBS, and concentrated.

### HIV-1 neutralization assay

Antibody neutralization was assessed by the single-round infection assay of TZM-bl cell with HIV-1 Env pseudovirus^60–62^. Mutant Envs or antibody mutations were generated by TagMaster Site-Directed Mutagenesis Kit (GMBiosciences, Frederick, MD) and tested along with the corresponding wild-type virus. Briefly, 50 μL of antibody-virus mixture was incubated at 37 °C for 30 min in duplicate wells before the addition of TZM-bl cells. To keep assay conditions constant, sham medium was used in place of antibody in control wells. Infection levels were determined in 2 days with Bright-Glo luciferase assay system (Promega, Madison, WI). Neutralization curves were fit with a 5-parameter asymmetric model or sigmoidal dose response with variable slope and with a constraint of bottom constant to 0 in Prism 9.0 (GraphPad Software, La Jolla, CA) and the data were plotted as means with standard error of the mean (SEM). The antibody concentration required to inhibit infection by 50% was reported as IC_50_.

### Enzyme-linked immunosorbent assay (ELISA)

Immulon 4 HBX plates were coated with HIV-1 BG505 DS-SOSIP or other antigens at 2 μg mL^−1^ in PBS and incubated at 4 °C overnight. Plates were blocked with 3% BSA in PBS for 2 h. M4008_N1 mAb or its variants was added at a starting concentration of 10 μg mL^−1^ in PBS with three-fold serial dilutions and incubated for 2 h. A 1:2000 dilution in PBS with 0.05% Tween-20 (PBST) of alkaline phosphatase-conjugated Goat anti-Human IgG (Southern Biotech) was added, and the plates were incubated for 1 h. Phosphatase substrate (Sigma) of 1 mg mL^−1^ in 10% diethanolamine was added and incubated in the dark for 30 min, and plates were read at 405 nm on a Biotek plate reader. Plates were washed three times in between each step with PBST. All steps aside from coating were conducted at room temperature, with a wash volume of 300 μL per well, blocking volume of 200 μL per well, and 100 μL per well for all other steps. Assays were performed in duplicate wells for three independent experiments. Binding curves were fit with a 5-parameter asymmetric model (GraphPad Software, La Jolla, CA) and the data were plotted as means with SEM.

### M4008_N1 Fab preparation

To generate M4008_N1 Fab, papain (Worthington) was activated with 20 mM cysteine and incubated with purified M4008_N1 IgG at a weight ratio of 1:10 (papain:IgG) in 20 mM Tris pH 7.5 with 150 mM NaCl for 1.5 h at 37 °C. To isolate Fab from Fc and undigested IgG, the reaction mixture was passed through a HiTrap Protein A HP column (GE Healthcare) in an equilibration buffer of 20 mM Tris pH 7.5 with 150 mM NaCl. Fab collected from flow-through was further purified by size-exclusion chromatography (SEC) on a HiLoad 16/60 Superdex 200 prep grade 120 mL column (GE Healthcare). Fab purity was analyzed on a 12% SDS-PAGE gel.

### Negative stain EM

For negative stain EM, a 3 μL aliquot of the complex sample at a concentration of 0.01 μg μL^−1^ was applied onto a glow-discharged carbon-coated grid (FCF400-CU, Electron Microscopy Sciences, EMS), blotted with a piece of filter paper (Whatman 1) and stained using 0.75% (w/v) uranyl formate (EMS) for 30 s. The images were collected on an FEI Talos L120C TEM at 120 kV coupled with a Gatan OneView camera. Each image was acquired in a low-dose mode at magnification of ×73,000 resulting in a pixel size of 2.0 Å, using a dose rate of ~24 e Å^−2^. All single particle processing was performed using cryoSPARC^63^.

### Cryo-EM sample preparation

To prepare a complex sample for cryo-EM, a 6-fold molar excess of M4008_N1 Fab was incubated with BG505 DS-SOSIP at room temperature for 1 h. The complex solution was concentrated to ~2 mg mL^−1^ using a 100 kDa cutoff concentrator (Amicon Ultra, Millipore), which also removed excess Fab. All complex samples for cryo-EM were verified for purity and homogeneity with negative stain EM as described above. To prevent protein aggregation and to obtain an optimal ice layer on the grid, n-dodecyl-β-d-maltopyranoside (DDM) solution was added to a final concentration of 0.085 mM prior to freezing. To prepare sample grids, a 3 μL aliquot of each sample was applied onto a C-Flat 1.2/1.3 grid (EMS), which had been freshly glow-discharged for 25 s at 15 mA using a PELCO easiGLOW (TED PELLA). The sample was plunged into liquid ethane using Vitrobot^TM^ Mark IV (FEI) after blotting with filter paper (Whatman) for 3 s at 4 °C under 100% relative humidity.

### Cryo-EM data collection

Micrographs were collected on a Titan Krios (FEI) operated at 300 kV with a K2 direct electron detector (Gatan), using a GIF-Quantum energy filter with a 15 eV slit width. Leginon^64^ was used for ice thickness targeting and automated data collection. Each micrograph was collected at ×130,000 nominal magnification resulting in a calibrated pixel size of 1.048 Å, using a dose rate of 7.26 e^−^ Å^−2^ s^−1^ with a total exposure of 8 s and an accumulated dose of 58.07 e^−^ Å^−2^ over 40 frames. A total of 7761 micrographs were collected at a nominal defocus range of 1.0–1.7 μm.

### Data processing and refinement

Micrograph frames were aligned using MotionCor2^65^. The contrast-transfer-function (CTF) estimation was performed with CTFFIND4^66^ using Appion^67^ for real-time pre-processing. Particle picking was trained in Warp^68^. Further processing including 2D classification, 3D refinement and map sharpening was performed using cryoSPARC^63^. Briefly, a total of 1,300,436 Warp extracted particles were sorted by reference-free 2D classification along with subset selection. A total of 346,795 selected particles from the classes that exhibited Fab-bound trimer features were further sorted into four classes by 3D heterogeneous refinement using an Ab-initio model low-pass filtered to 30 Å without importing symmetry. The 281,313 particles (81%) belonging to the classes that were apparently fully occupied trimers were combined and subsequently subjected to 3D non-uniform refinement. The initial 3D reconstruction map was obtained without imposing symmetry to a resolution of 3.23 Å. Base on visual inspection, the reconstruction map exhibited a 3-fold symmetry despite some slight deviation at the region corresponding to the constant domain of M4008_N1 Fab. Therefore, the same particles were refined with C3 symmetry imposed. The overall resolution of the final map was determined to 3.24 Å, based on the gold-standard Fourier shell curve (FSC) using a correlation cut-off of 0.143^69^ (Supplementary Fig. 1).

### Model building

To create a model for M4008_N1 Fab, a homologous Fab (PDB ID 4OAW^70^) was used as an initial homology model based on multiple amino acid sequences searching in SWISS-MODEL^71^. To generate a complex model, the gp120 and gp41 subunits from a previous DS-SOSIP structure (PDB ID 5U1F^20^) were used as reference models and fitted separately into the final reconstructed cryo-EM density map of M4008_N1 Fab/DS-SOSIP complex using UCSF Chimera^72^. Due to weak densities observed at the regions corresponding to the constant domains of M4008_N1, only the variable domains of heavy and light chains were used to fit separately into the corresponding density regions. The initial complex model was manually inspected and adjusted in COOT^73^, particularly at the regions that were spatially clashed or truncated in the reference models. The initial refinement was performed using Phenix^74^ by a single round of rigid body refinement, morphing, and simulating annealing, followed by multiple iterations of model building and real space refinement using COOT and Phenix, respectively. To model glycans, the corresponding regions of potential N-linked glycosylation sites on the cryo-EM map were interpreted. For the region observed with glycan-related densities, an N-linked NAG-NAG-BMA was built using the carbohydrate module in COOT^75^, followed by subsequent modification to match map densities. The final refinement was carried out by real space and B-factor refinement with secondary structure restraints using Phenix. Model validation was done using MolProbity^76^. The interface and buried surface area (BSA) and the potential hydrogen bond were calculated using PISA^77^. The Cα RMSD was calculated using the script available in the PyMOL wiki. All the figures were generated using UCSF Chimera or PyMOL^78^.

### Reporting summary

Further information on research design is available in the Nature Research Reporting Summary linked to this article.

## Supplementary information

Supplementary Information

Description of Additional Supplementary Files

Supplementary Data 1

Reporting summary

## Data Availability

The cryo-EM map and coordinates for the M4008_N1-bound BG505 DS-SOSIP trimer have been deposited in the Electron Microscopy Data Bank (EMBD) and the Protein Data Bank (PDB) with the accession codes EMD-24362 and 7RAI, respectively. Raw data of graphs are provided in Suppl. Data 1.
