# Supplementary material for: A site of vulnerability at V3 crown defined by HIV-1 bNAb M4008_N1
Source: Nat Commun. 2021 Nov 9;12:6464. doi: 10.1038/s41467-021-26846-z (PMC8578649; doi:10.1038/s41467-021-26846-z)
Supplement: Supplementary file 1 — Supplementary Information [file 41467_2021_26846_MOESM1_ESM.pdf]

Supplementary Information

A site of vulnerability at V3 crown defined by HIV-1 bNAb M4008_N1

Kun-Wei Chan[1], Christina C. Luo[1], Hong Lu[2], Xueling Wu[2], and Xiang-Peng Kong[1,*]

[1]Department of Biochemistry and Molecular Pharmacology, NYU Grossman School of Medicine, New York, NY 10016, USA

[2]Aaron Diamond AIDS Research Center, Columbia University Vagelos College of Physicians and Surgeons, New York, NY 10032, USA

[*]correspondence: xiangpeng.kong@med.nyu.edu

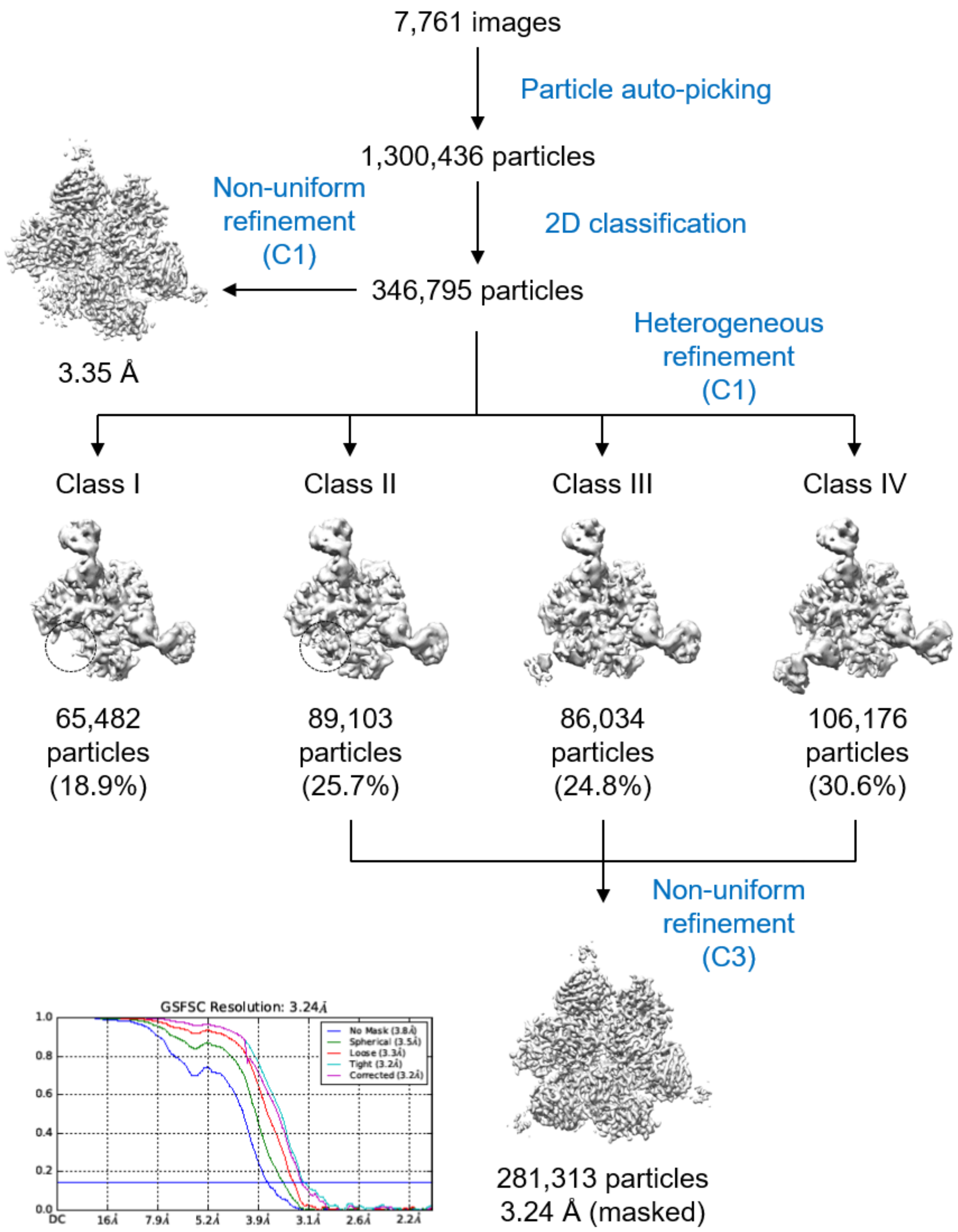

Supplementary Fig. 1 Cryo-EM data processing. Data processing was performed by cryoSPARC[1]. After the heterogeneous refinement, particles belonging to the groups of fully occupied complexes (81% of selected complex particles) were used for the final refinement. The dashed-line circles in Classes I and II indicate one of the M4008_N1 binding sites. Densities at the site of Class II revealed that particles in Class II were fully occupied, whereas lack of densities at the site of Class I suggested that particles in Class I were partially bound with two M4008_N1 Fabs. Thus, only Classes II-IV were included in the 3D reconstruction.

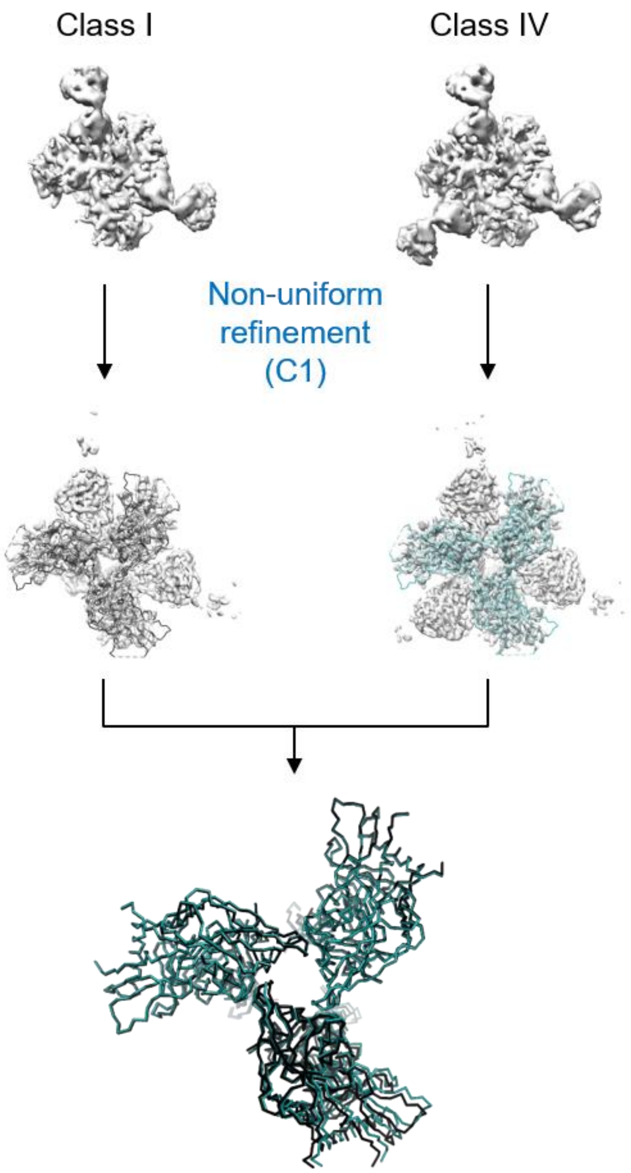

Supplementary Fig. 2 Superimposition of the fully occupied trimer and the partially occupied trimer. The 3D reconstruction maps belonging to the partially occupied trimer (Fab:Env trimer ratio 2:1, Class I in **Supplementary Fig. 1**) and the fully occupied trimer (3:1, Class IV) were processed separately, and gp120 and gp41 were docked into them. The trimer model of the partially occupied one is colored black, while that of the fully occupied one is colored cyan.

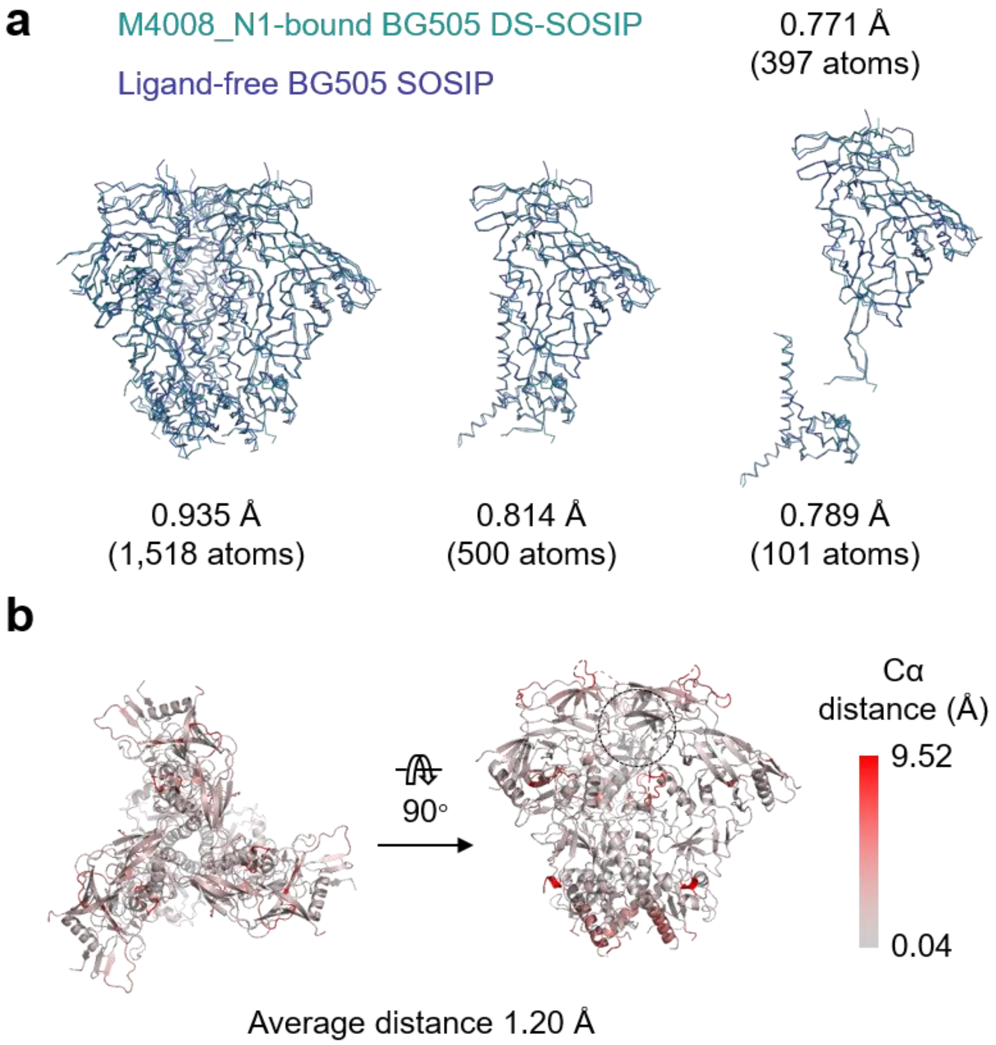

Supplementary Fig. 3 Structural comparison of M4008_N1-bound trimer with a ligand-free trimer. a Superimposition of the M4008_N1-bound trimer and the ligand-free trimer (PDB ID 4ZMJ) revealed that the conformation of the trimer as well as each structural component did not change upon M4008_N1 binding. The Cα RMSD was calculated by PyMOL[2] with 3 cycles of outlier rejection. **b** The M4008_N1-bound trimer is colored by its differences of Cα in distance compared to the ligand-free trimer. The white color indicates less different regions, while the red color indicates the most different regions or regions that were not defined. The result revealed that the most changes were at the variable regions (e.g., V1 and V2 loops). The M4008_N1 binding site is indicated by a black dashed-line circle.

a

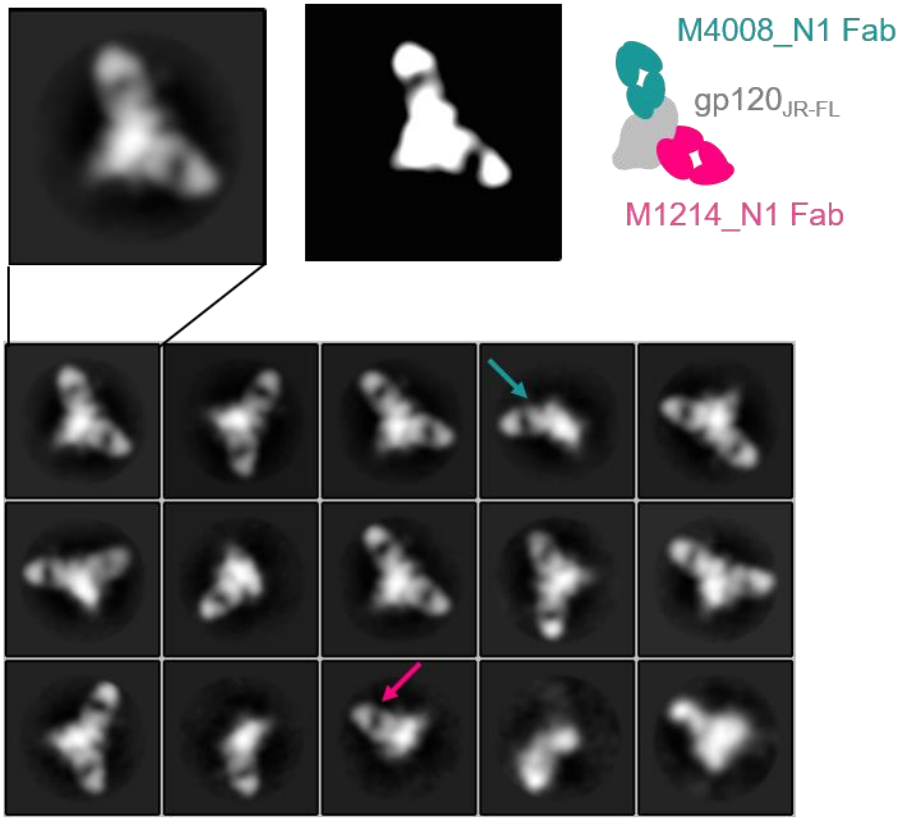
M4008_N1 Fab
gp120JR-FL
M1214_N1 Fab

b

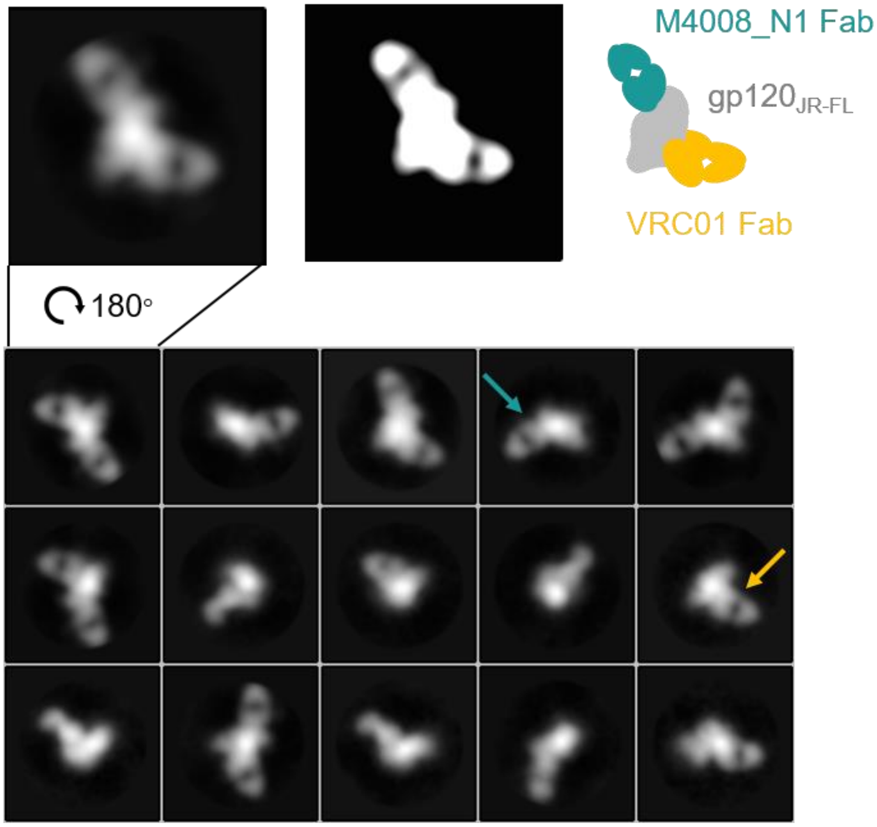
M4008_N1 Fab
gp120JR-FL
VRC01 Fab
180°

Supplementary Fig. 4 A negative staining visualization of the ternary complex of JR-FL gp120 with the Fabs of bNAbs M4008_N1 and M1214_N1 or VRC01. These data revealed that M4008_N1 binding to JR-FL gp120 did not affect **a** V2V5 corridor bNAb M1214_N1 Fab or **b** CD4bs bNAb VRC01 Fab binding, inferring that binding of M4008_N1 does not alter the conformation of V2V5 corridor or CD4bs.

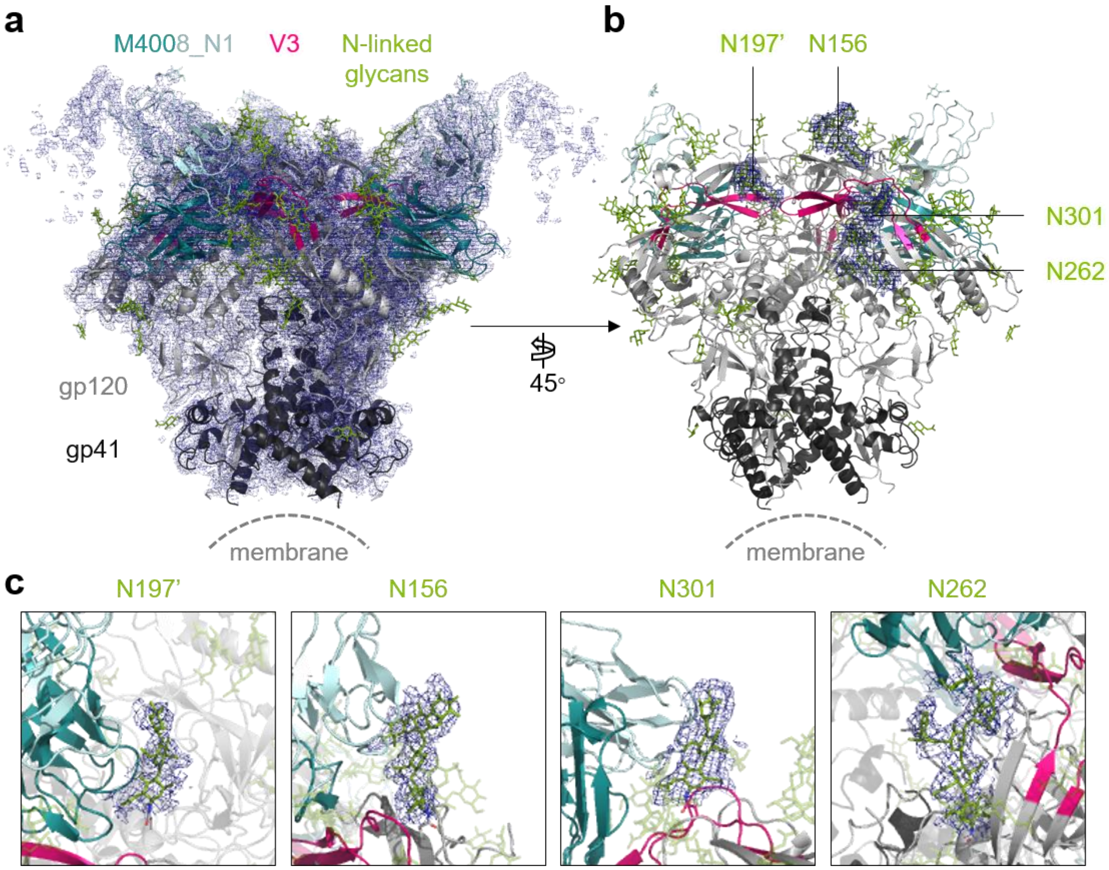

Supplementary Fig. 5 M4008_N1 binding site-surrounding glycans. a Cryo-EM density map (blue mesh) and the final structural model of the M4008_N1/DS-SOSIP complex. We observed glycan densities for 18 of the 28 potential N-linked glycosylation sites of each gp120/gp41 protomer and built glycan residue(s) into them (green sticks). **b** Four key glycans, glycans N156, N262 and N301 from the primary gp120 and N197 from the neighboring gp120, surrounding the M4008_N1 binding site are highlighted with their densities. For clarity, the front M4008_N1 Fab was removed. **c** These glycans are shown individually.

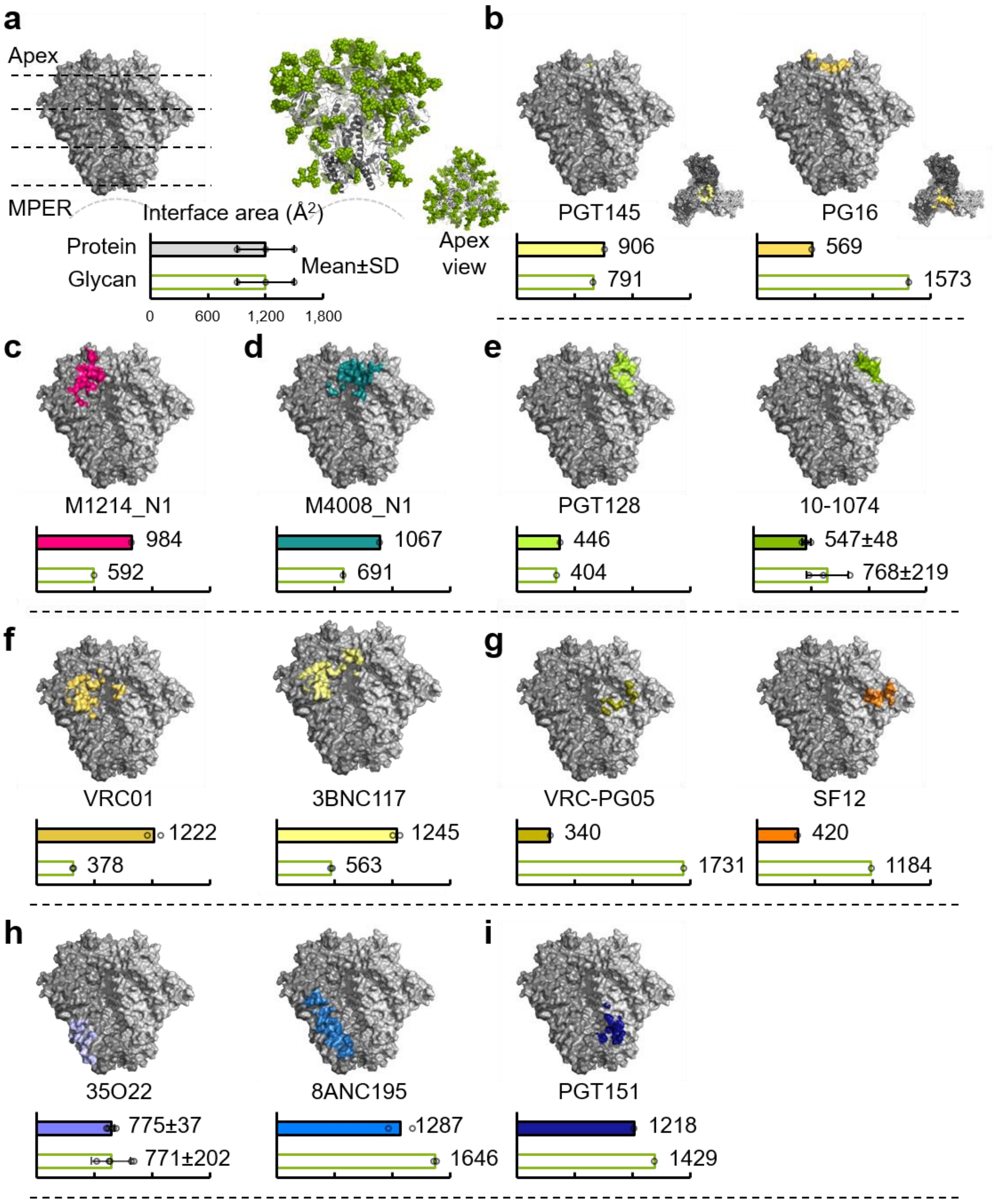

Supplementary Fig. 6 Currently known vulnerable sites on HIV-1 Env. a A model of the HIV-1 Env trimer. **b-i** The vulnerable sites defined by some of the currently known HIV-1 bNAbs, except the MPER region, are individually displayed on the HIV-1 Env trimer model from distal region to MPER region, including V1V2 apex (**b**), V2V5 corridor (**c**), V3 crown (**d**), V3 base glycan supersite (**e**; n = 3 for 10-1074), CD4bs (**f**), silent face (**g**), gp120/gp41 interface (**h**; n = 6 for 35O22), and fusion peptide regions (**i**). The interface areas were calculated using

PDBePISA (https://www.ebi.ac.uk/pdbe/pisa/) based on the published coordinates (**Supplementary Table 9** and **Supplementary Data 1**) and displayed as bar charts with a filled or outlined bar for protein or glycan interface area, respectively, and individual values depicted with an open circle. For bNAbs with n = 2 models, data are represented as means; for bNAbs with n ≥ 3 models, data are represented as means ± standard deviation (SD).

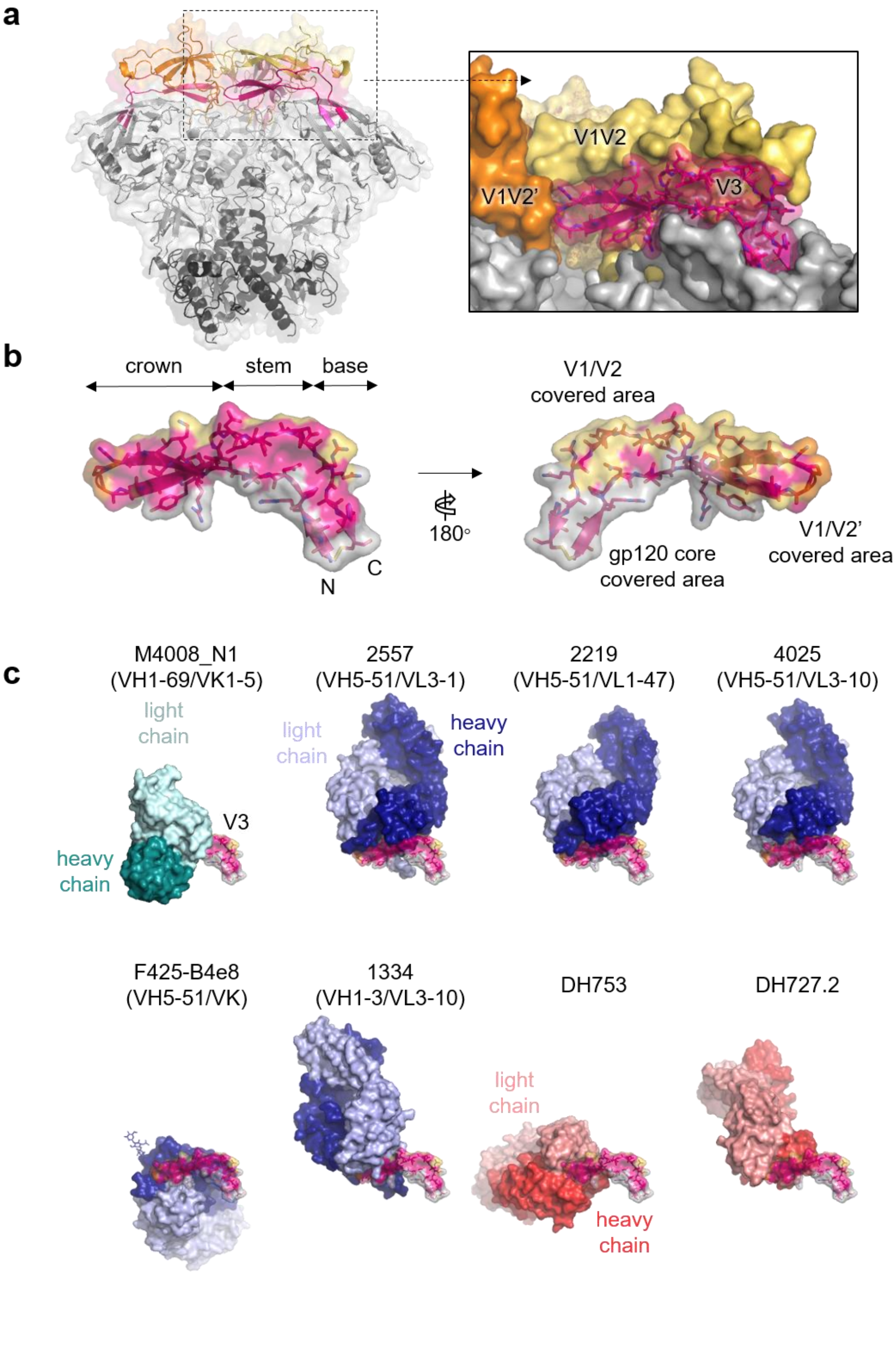
a
V1V2
V1V2'
V3
b
crown
stem
base
N
C
180°
V1/V2
covered area
gp120 core
covered area
V1/V2'
covered area
c
M4008_N1
(VH1-69/VK1-5)
light
chain
V3
heavy
chain
2557
(VH5-51/VL3-1)
light
chain
heavy
chain
2219
(VH5-51/VL1-47)
4025
(VH5-51/VL3-10)
F425-B4e8
(VH5-51/VK)
1334
(VH1-3/VL3-10)
DH753
light
chain
heavy
chain
DH727.2

Supplementary Fig. 7 Comparison of the epitopes of V3 crown mAbs with that of bNAb M4008_N1. a The V3 region (in pink) of the primary gp120 in the closed form Env trimer is shown in sticks with a semi-transparent surface. The V1V2 domains of the primary gp120 and the neighboring gp120 are colored yellow and orange, respectively. **b** Different views of the selected V3 region in panel **a**. The protected regions on V3 are highlighted with different colors: those shielded by V1V2 are colored yellow and orange while those shielded by the gp120 core are colored gray. For clarity, the surrounding glycans were removed and their masking effects ignored. **c** The V3 crown binding modes of some previously characterized V3 crown antibodies are compared with that of M4008_N1. Note that the V3 crown binding of these mAbs is not compatible with the closed form of the Env trimer, unlike that of M4008_N1. The V3 crown mAbs in panel **c** include M4008_N1 (this paper), 2557 (PDB ID 3MLR (https://doi.org/10.2210/pdb3mlr/pdb)), 2219 (2B1A (https://doi.org/10.2210/pdb2b1a/pdb)), 4025 (3UJJ (https://doi.org/10.2210/pdb3ujj/pdb)), F425-B4e8 (2QSC (https://doi.org/10.2210/pdb2qsc/pdb)), 1334 (6DB7 (https://doi.org/10.2210/pdb6db7/pdb)), DH753 (6MNR (https://doi.org/10.2210/pdb6mnr/pdb)) and DH727.2 (6MNQ (https://doi.org/10.2210/pdb6mnq/pdb)).

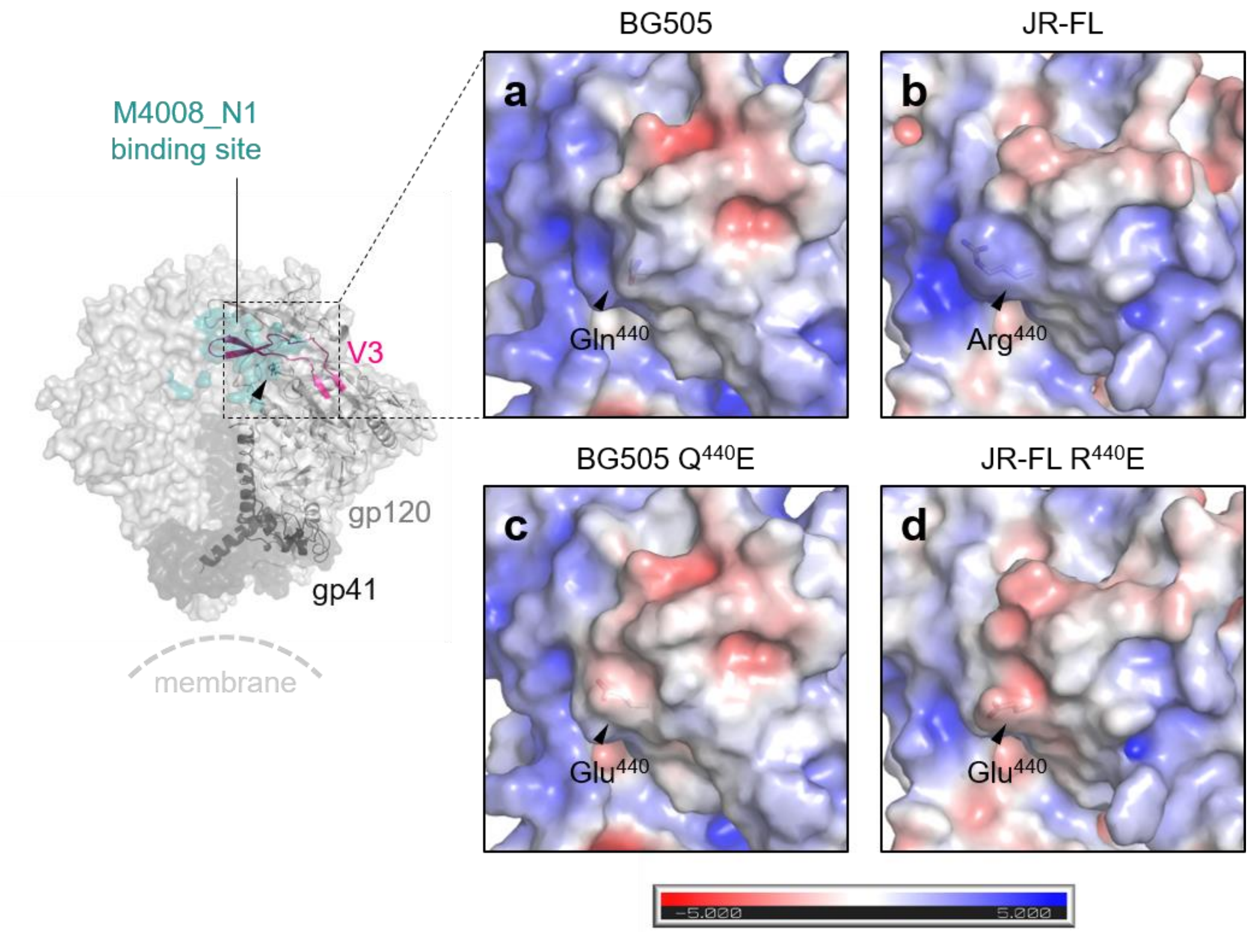

Supplementary Fig. 8 Effect of residue 440 on M4008_N1 neutralization sensitivity. a-d Electrostatic potential surfaces around residue 440 (arrowhead) on strains BG505 (**a**), JR-FL (**b**), BG505 Q^{440}E (**c**), and JR-FL R^{440}E (**d**) are shown using PyMOL[2] in the range of -5 to +5 (units $k_B T/e_c$) with the red indicating negative and blue positive potentials. Structural models of BG505 Q^{440}E and JR-FL R^{440}E were created with SWISS-MODEL[3] using BG505 DS-SOSIP (this manuscript, chain A) and JR-FL SOSIP (PDB ID 5FYK (https://doi.org/10.2210/pdb5fyk/pdb), chain G), respectively, as a template.

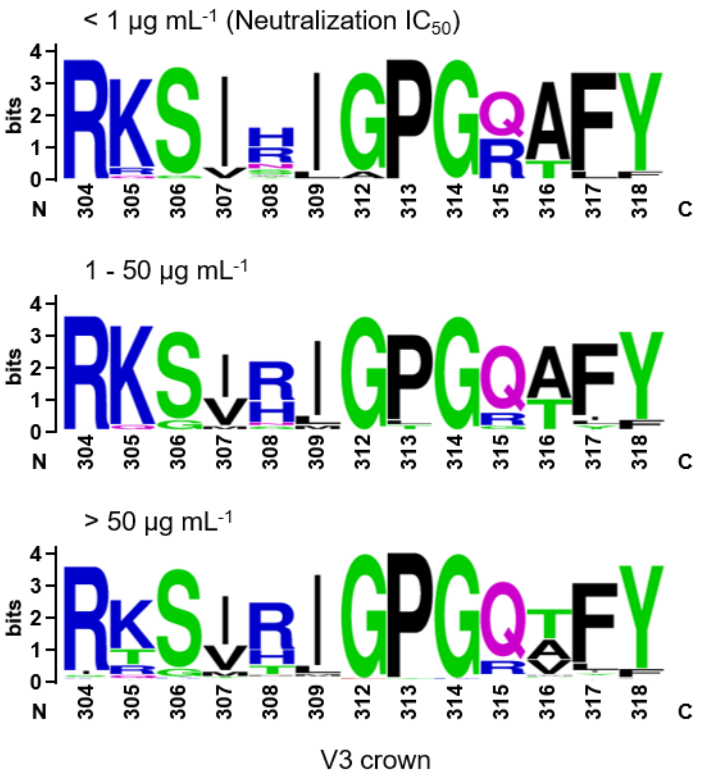

Supplementary Fig. 9 Sequence logos of the V3 crown region from the strains (n=120) tested in the previous neutralization analysis[4]. The overall height of the stack indicates the sequence conservation at that position, while the height of the symbols within the stack indicates the relative frequency of each amino acid at that position. Sequence logos were generated using Weblogo (https://weblogo.berkeley.edu/logo.cgi).

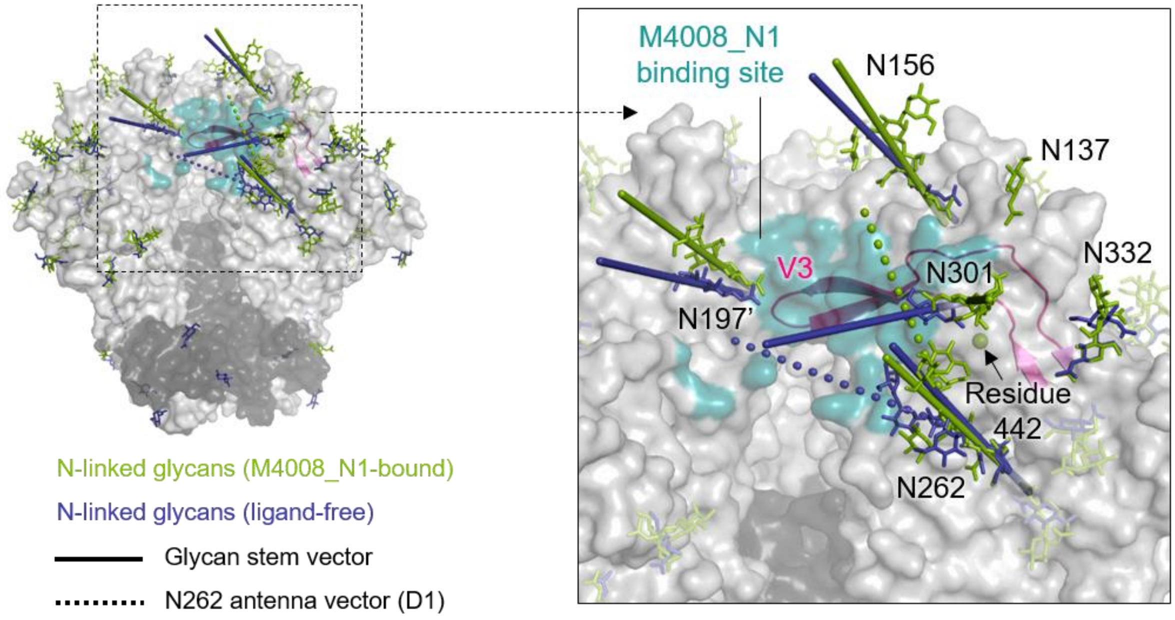

Supplementary Fig. 10 Insertion of glycan at position 442 might restrict the movement of glycan N301 to adapt to M4008_N1 binding. The M4008_N1 binding site and the conformational change of surrounding glycans upon M4008_N1 binding are presented as in **Fig. 2b**. Insertion of glycan at position 442 (green dot), which is located between glycans N262, N301 and N332, might form spatial constraints, particularly against glycan 301, to adapt to M4008_N1 binding.

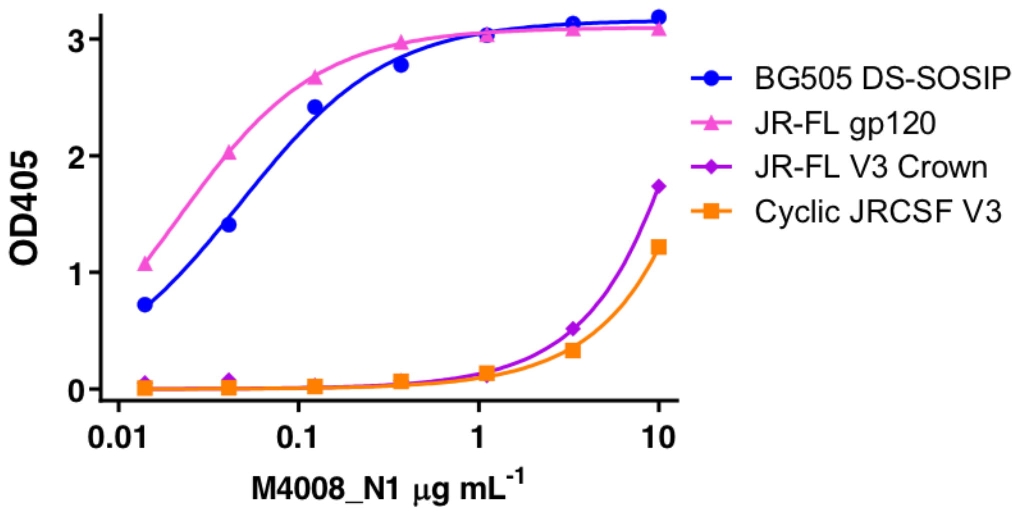

Supplementary Fig. 11 ELISA analysis of M4008_N1 binding to different V3 antigens. M4008_N1 binding was assayed against trimeric stabilized Env (BG505 DS-SOSIP), monomeric gp120 (JR-FL gp120), a linear V3 crown peptide (JR-FL V3 Crown), and a disulfide-bridged cyclic V3 peptide (Cyclic JRCSF V3).

	**M4008_N1 Fab/BG505 DS-SOSIP**<br>PDB ID 7RAI, EMD-24362
**Data collection and processing**	
Microscope	FEI Titan Krios
Camera	Gatan K2
Magnification	130,000
Voltage (kV)	300
Electron exposure (e^{-} Å^{-2})	58.07
Defocus range (μm)	-1.0 to -1.7
Pixel size (Å)	1.048
Initial particle (no.)	346,795
Final particle (no.)	281,313
Symmetry imposed	C3
Map resolution (masked, Å)	3.24
FSC threshold	0.143
Map sharpening *B* factor (Å^{2})	-137.1
**Refinement**	
Model composition	
Non-hydrogen atoms (no.)	20652
Protein residues (no.)	2424
Sugar residues (no.)	132
RMSD	
Bond lengths (Å)	0.007
Bond angles (°)	0.692
Validation	
Clashscore	9.88
Poor rotamers (%)	7.69
Ramachandran statistics (%)	
Favored	91.29
Allowed	7.69
Outliers	0.00

Supplementary Table 1 Cryo-EM data collection, processing, and refinement statistics

BG505	Mutation	IC_{50} μg mL^{-1}	Ratio to WT	Fold change
WT		1.07	1.00	1
ΔN137	N137A	0.66	0.62	1.6
ΔN156	N156Q	0.61	0.57	1.8
ΔN197	N197D	0.05	0.05	21
ΔN301	T303A	0.03	0.03	35

JR-FL	Mutation	IC_{50} μg mL^{-1}	Ratio to WT	Fold change
WT		0.007	1.00	1
ΔN156	N156Q	0.03	4.00	4
D197N	D197N	0.23	32.86	33
ΔN301	T303A	0.0002	0.03	35

Supplementary Table 2 Neutralization analyses of M4008_N1 against different glycan-modified BG505 and JR-FL variants.

Gp120 residues	Gp120 regions	BSA(Å^2)	Level of conservation
K65	C1	21.3	0.993
T163	V2, strand b	22.4	0.868
E164	V2, loop b/c	25.9	0.280
Q170	V2, strand c	26.8	0.420
V172	V2, strand c	14.1	0.236
N197’	V1V2 stem	32.9	0.967
T198’	V1V2 stem	10.5	0.828
P206	C2	33.3	0.983
K207	C2	67.8	0.984
T303	V3	44.0	0.903
R304	V3	96.1	0.885
K305	V3	43.3	0.531
S306	V3	69.3	0.663
R308	V3	102.5	0.306
G314	V3	16.1	0.961
A316	V3	15.7	0.449
Y318	V3	44.2	0.846
D321a	V3	34.7	0.342
I323	V3	80.7	0.785
G324	V3	16.6	0.970
D368’	C3, CD4 binding loop	38.3	0.990
Q428’	C4	40.1	0.955
I430’	C4	95.5	0.831
Q440	C4	21.3	0.168
Geometric mean			0.637

Supplementary Table 3. Key contacts of M4008_N1 on the Env trimer and levels of conservation among 7,094 global Env sequences (version year 2018) retrieved from the Los Alamos HIV sequence database (http://www.hiv.lanl.gov). Residues with an apostrophe indicate those from the neighboring gp120. Buried surface area (BSA) was calculated using PDBePISA (https://www.ebi.ac.uk/pdbe/pisa/). Only residues with BSA > 10 Å^2 are listed. Level of conservation was calculated using an entropy scoring method implemented in the R package Bio3D.

BG505	IC_{50} μg mL^{-1}	Ratio to WT	Fold change
WT	1.07	1.0	1.0
$E^{164}S$	0.332	0.3	3.2
$R^{304}S$	>50	>47	>47
$K^{305}Q$	0.373	0.3	2.9
$S^{306}K$	>50	>47	>47
$I^{307}T$	0.765	0.7	0.7
$R^{308}G$	0.235	0.2	4.6
$D^{321a}R$	1.05	1.0	1.0
$Q^{440}E$	2.87	2.7	2.7

JR-FL	IC_{50} μg mL^{-1}	Ratio to WT	Fold change
WT	0.006	1.0	1
$S^{164}E$	0.006	1.0	1
$R^{304}S$	2.84	474	474
$K^{305}Q$	0.078	13.0	13
$S^{306}K$	>50	>8333	>8333
$I^{307}T$	0.086	14.3	14
$H^{308}G$	0.001	0.17	6
$E^{321a}R$	0.009	1.5	1.5
$R^{440}E$	>50	>8333	>8333

Supplementary Table 4 Neutralization analyses of M4008_N1 against different BG505 and JR-FL variants.

	**Neutralization**		**ELISA**	
**M4008_N1**	**IC_{50} μg mL^{-1}**	**Ratio to WT**	**IC_{50} μg mL^{-1}**	**Ratio to WT**
WT	0.42	1	0.01	1
$F^{H100}S$	0.67	1.6	0.01	1.1
$S^{H100a}A$	0.68	1.6	0.03	2.1
$C^{H100b}S$	27.18	65	0.51	40.9
$E^{H100c}R$	48.46	115	>3	>240
$M^{H100d}A$	>50	>120	>3	>240
$D^{H100e}A$	>50	>120	>3	>240

Supplementary Table 5 Neutralization and ELISA analyses of M4008_N1 CDR H3 variants against BG505.

VH	FWR1	CDR1	FWR2	CDR2	FWR3	FWR	CDR	Total
No. of residues	30	5	14	17	32	76	22	98
No. of mutated	7	5	1	7	13	21	12	33
Mutation rate (%)						27.6	54.5	33.7

VL	FWR1	CDR1	FWR2	CDR2	FWR3	FWR	CDR	Total
No. of residues	23	11	15	7	32	70	18	88
No. of mutated	7	6	3	5	8	18	11	29
Mutation rate (%)						25.7	61.1	33.0

Supplementary Table 6 Number of somatically mutated residues in the variable regions of M4008_N1. CDR H3 and CDR L3 were not included.

M4008_N1	IC_{50} μg mL^{-1}	Ratio to WT
WT	0.01	1
$F^{H28}T$	0.02	1.6
$Y^{H31}S$	0.04	2.9
$D^{H54}F$	0.02	1.9
$R^{L32}W$	0.12	9.3
$R^{L50}D$	0.02	1.8

Supplementary Table 7 ELISA analysis of M4008_N1 SHM revertants against BG505.

**Antigens**	**IC_{50} μg mL^{-1}**
BG505 DS-SOSIP	0.05
JR-FL gp120	0.02
JR-FL V3 Crown	7.42
Cyclic JRCSF V3	10.22

Supplementary Table 8 ELISA analysis of M4008_N1 binding to different antigens.

bNAbs	PDB ID	
**V1V2 apex**		
PGT145	5V8L	https://doi.org/10.2210/pdb5v8l/pdb
PG16	6ULC	https://doi.org/10.2210/pdb6ulc/pdb
**V2V5 corridor**		
M1214_N1	6VY2	https://doi.org/10.2210/pdb6vy2/pdb
**V3 crown**		
M4008_N1	This paper	
**V3 base glycan**		
10-1074	5T3X	https://doi.org/10.2210/pdb5t3x/pdb
	5T3Z	https://doi.org/10.2210/pdb5t3z/pdb
	6OKP	https://doi.org/10.2210/pdb6okp/pdb
PGT122	4TVP	https://doi.org/10.2210/pdb4tvp/pdb
	5D9Q	https://doi.org/10.2210/pdb5d9q/pdb
	5FYJ	https://doi.org/10.2210/pdb5fyj/pdb
	5FYK	https://doi.org/10.2210/pdb5fyk/pdb
	5FYL	https://doi.org/10.2210/pdb5fyl/pdb
PGT124	6IEQ	https://doi.org/10.2210/pdb6ieq/pdb
	6MCO	https://doi.org/10.2210/pdb6mco/pdb
	6MDT	https://doi.org/10.2210/pdb6mdt/pdb
PGT128	6OPA	https://doi.org/10.2210/pdb6opa/pdb
**CD4bs**		
VRC01	5FYJ	https://doi.org/10.2210/pdb5fyj/pdb
	5FYK	https://doi.org/10.2210/pdb5fyk/pdb
3BNC117	5V8L	https://doi.org/10.2210/pdb5v8l/pdb
	5V8M	https://doi.org/10.2210/pdb5v8m/pdb
IOMA	5T3X	https://doi.org/10.2210/pdb5t3x/pdb
	5T3Z	https://doi.org/10.2210/pdb5t3z/pdb
**silent face**		
VRC-PG05	6BF4	https://doi.org/10.2210/pdb6bf4/pdb
SF12	6OKP	https://doi.org/10.2210/pdb6okp/pdb

Supplementary Table 9 PDB models used to calculate the interface area of individual vulnerable sites including those in **Supplementary Fig. 6**.

bNAbs	PDB ID	
**gp120/gp41 interface**		
35O22	4TVP	https://doi.org/10.2210/pdb4tvp/pdb
	5FYJ	https://doi.org/10.2210/pdb5fyj/pdb
	5FYK	https://doi.org/10.2210/pdb5fyk/pdb
	5FYL	https://doi.org/10.2210/pdb5fyl/pdb
	6IEQ	https://doi.org/10.2210/pdb6ieq/pdb
	6OPA	https://doi.org/10.2210/pdb6opa/pdb
8ANC195	5CJX	https://doi.org/10.2210/pdb5cjx/pdb
	6NQD	https://doi.org/10.2210/pdb6nqd/pdb
**fusion peptide**		
PGT151	6OLP	https://doi.org/10.2210/pdb6olp/pdb

Supplementary Table 9 PDB models used to calculate the interface area of individual vulnerable sites including those in **Supplementary Fig. 6**. *Continued.*

M4008_N1 Mutation	Forward primer	Reverse primer
$F^{H100}S$	gtccatctcgcaactgctccctgcgcactgaagt	acttcagtgcgcagggagcagttgcgagatggac
$S^{H100a}A$	gagtccatctcgcaagcgaaccctgcgcactg	cagtgcgcagggttcgcttgcgagatggactc
$C^{H100b}S$	gtccatctcgctactgaaccctgcgcact	agtgcgcagggttcagtagcgagatggac
$E^{H100c}R$	gcagggttcagttgcaggatggactcgggacc	ggtcccgagtccatcctgcaactgaaccctgc
$M^{H100d}A$	caaatggtcccgagtccgcctcgcaactgaaccctg	cagggttcagttgcgaggcggactcgggaccatttg
$D^{H100e}A$	gatcaaatggtcccgaggccatctcgcaactgaac	gttcagttgcgagatggcctcgggaccatttgatc
$F^{H28}T$	ctgcaaagactcaggaggcactttcccttatgctggcttc	gaagccagcataagggaaagtgcctcctgagtctttgcag
$Y^{H31}S$	aagactcaggaggctttttccctagtgctggcttccg	cggaagccagcactagggaaaaagcctcctgagtctt
$D^{H54}F$	ggaggcgtcattcctgcgttcgggacaaaacattatgc	gcataatgttttgtcccgaacgcaggaatgacgcctcc
$R^{L32}W$	cggaccagtcaaggtgttggcaattggttggcctggtatc	gataccaggccaaccaattgccaacaccttgactggtccg
$R^{L50}D$	aaagccccctagactactcatttctgatgcctcaaatcga	tcgatttgaggcatcagaaatgagtagtctagggggcttt

Supplementary Table 10 Primers for the site-directed mutagenesis of M4008_N1 to create variants used for assays in **Fig. 4** and **Fig. 5**.

Supplementary references

1. Punjani, A., Rubinstein, J.L., Fleet, D.J. & Brubaker, M.A. cryoSPARC: algorithms for rapid unsupervised cryo-EM structure determination. *Nat Methods* **14**, 290-296 (2017).
2. Schrodinger, LLC. The PyMOL Molecular Graphics System, Version 1.8. (2015).
3. Waterhouse, A., Bertoni, M., Bienert, S., Studer, G., Tauriello, G., Gumienny, R., Heer, F.T., de Beer, T.A.P., Rempfer, C., Bordoli, L., Lepore, R. & Schwede, T. SWISS-MODEL: homology modelling of protein structures and complexes. *Nucleic Acids Res* **46**, W296-W303 (2018).
4. Jia, M., Liberatore, R.A., Guo, Y., Chan, K.W., Pan, R., Lu, H., Waltari, E., Mittler, E., Chandran, K., Finzi, A., Kaufmann, D.E., Seaman, M.S., Ho, D.D., Shapiro, L., Sheng, Z., Kong, X.P., Bieniasz, P.D. & Wu, X. VSV-Displayed HIV-1 Envelope Identifies Broadly Neutralizing Antibodies Class-Switched to IgG and IgA. *Cell Host Microbe* **27**, 963-975 e965 (2020).
