# Supplementary material for: A site of vulnerability at V3 crown defined by HIV-1 bNAb M4008_N1
Source: Nat Commun. 2021 Nov 9;12:6464. doi: 10.1038/s41467-021-26846-z (PMC8578649; doi:10.1038/s41467-021-26846-z)
Supplement: Supplementary file 2 — Description of Additional Supplementary Files [file 41467_2021_26846_MOESM2_ESM.pdf]

Description of Additional Supplementary Files

Supplementary Data 1:

Fig 2b: Conformational changes of M4008_N1 binding site-surrounding glycans upon M4008_N1 binding were calculated using atomic coordinates of a ligand-free SOSIP (PDB ID 4ZMJ (https://doi.org/10.2210/pdb4zmj/pdb)) and M4008_N1-bound DS-SOSIP (this manuscript), in which gp120 (chain G) of 4ZMJ was superimposed on the gp120 (chain A) of M4008_N1-bound trimer to obtain the aligned coordinates for comparison. Due to the lack of the glycan model, the conformational change of glycan N137 was represented by calculating the distance of the Asn^{137} Cα in the two structural models.

Supplementary Figure 6: The interface areas between various bNAbs of each vulnerable site and Env were calculated using PDBePISA (https://www.ebi.ac.uk/pdbe/pisa/) based on available structures. The total interface area between antibody and glycan of gp120 and/or gp41 was the sum of that between antibody and individual glycan residues. The ratio of protein to glycan (P/G) interface areas was also calculated for each bNAb, and mean ratios calculated for each vulnerable site.

Sequence alignment: Sequences of HIV gp120 from 120 strains tested previously (https://doi.org/10.1016/j.chom.2020.03.024), in which positions that harbor potential glycans around M4008_N1 binding site and key residues as listed in Supplementary Table 3 were aligned in tripeptide and single, respectively. Those tripeptides that show a potential N-linked glycan sequon (Asn-X-Ser/Thr) are highlighted in bold.
