# Supplementary material for: A site of vulnerability at V3 crown defined by HIV-1 bNAb M4008_N1
Source: Nat Commun. 2021 Nov 9;12:6464. doi: 10.1038/s41467-021-26846-z (PMC8578649; doi:10.1038/s41467-021-26846-z)
Supplement: Supplementary file 4 — Reporting summary [file 41467_2021_26846_MOESM4_ESM.pdf]

nature research

Corresponding author(s): Xiang-Peng Kong

Last updated by author(s): Oct 4, 2021

Reporting Summary

Nature Research wishes to improve the reproducibility of the work that we publish. This form provides structure for consistency and transparency in reporting. For further information on Nature Research policies, see our Editorial Policies and the Editorial Policy Checklist.

Statistics

For all statistical analyses, confirm that the following items are present in the figure legend, table legend, main text, or Methods section.

n/a	Confirmed	
☐	☒	The exact sample size (n) for each experimental group/condition, given as a discrete number and unit of measurement
☐	☒	A statement on whether measurements were taken from distinct samples or whether the same sample was measured repeatedly
☒	☐	The statistical test(s) used AND whether they are one- or two-sided<br>*Only common tests should be described solely by name; describe more complex techniques in the Methods section.*
☒	☐	A description of all covariates tested
☒	☐	A description of any assumptions or corrections, such as tests of normality and adjustment for multiple comparisons
☐	☒	A full description of the statistical parameters including central tendency (e.g. means) or other basic estimates (e.g. regression coefficient) AND variation (e.g. standard deviation) or associated estimates of uncertainty (e.g. confidence intervals)
☒	☐	For null hypothesis testing, the test statistic (e.g. F, t, r) with confidence intervals, effect sizes, degrees of freedom and P value noted<br>*Give P values as exact values whenever suitable.*
☒	☐	For Bayesian analysis, information on the choice of priors and Markov chain Monte Carlo settings
☒	☐	For hierarchical and complex designs, identification of the appropriate level for tests and full reporting of outcomes
☒	☐	Estimates of effect sizes (e.g. Cohen's d, Pearson's r), indicating how they were calculated

Our web collection on statistics for biologists contains articles on many of the points above.

Software and code

Policy information about availability of computer code

Data collection	Leginon v 3.5 was used for cryo-EM data collection
Data analysis	MotionCor2, CTFFIND4, Warp and CryoSparc v 2.15 were used for data processing. Phenix v 1.17.1, COOT v 0.8.9.2, UCSF Chimera v 1.13.1 and PyMOL v 2.3.3 were used for structural analysis. Graphpad Prism 8.0 and 9.0 were used for data analysis.

For manuscripts utilizing custom algorithms or software that are central to the research but not yet described in published literature, software must be made available to editors and reviewers. We strongly encourage code deposition in a community repository (e.g. GitHub). See the Nature Research guidelines for submitting code & software for further information.

Data

Policy information about availability of data

All manuscripts must include a data availability statement. This statement should provide the following information, where applicable:

- Accession codes, unique identifiers, or web links for publicly available datasets
- A list of figures that have associated raw data
- A description of any restrictions on data availability

The cryo-EM map and coordinates for the M4008_N1-bound BG505 DS-SOSIP trimer have been deposited in the Electron Microscopy Data Bank (EMDB) and Protein Data Bank (PDB) with the accession codes EMD-24362 and 7RAI, respectively. The data that support the findings of this study are provided with this paper or available from the corresponding author upon reasonable request.

Field-specific reporting

Please select the one below that is the best fit for your research. If you are not sure, read the appropriate sections before making your selection.

[x] Life sciences [ ] Behavioural & social sciences [ ] Ecological, evolutionary & environmental sciences

For a reference copy of the document with all sections, see nature.com/documents/nr-reporting-summary-flat.pdf

Life sciences study design

All studies must disclose on these points even when the disclosure is negative.

Sample size	The number of sample size used for statistical calculation has been mentioned in the main text or figure legends.
Data exclusions	No data was excluded from analyses.
Replication	Experiments were repeated and/or performed in triplicate unless otherwise noted.
Randomization	N/A
Blinding	Blinding was not relevant for this study.

Reporting for specific materials, systems and methods

We require information from authors about some types of materials, experimental systems and methods used in many studies. Here, indicate whether each material, system or method listed is relevant to your study. If you are not sure if a list item applies to your research, read the appropriate section before selecting a response.

Materials & experimental systems

n/a	Involved in the study	
[ ]	[x]	Antibodies
[ ]	[x]	Eukaryotic cell lines
[x]	[ ]	Palaeontology and archaeology
[x]	[ ]	Animals and other organisms
[x]	[ ]	Human research participants
[x]	[ ]	Clinical data
[x]	[ ]	Dual use research of concern

Methods

n/a	Involved in the study	
[x]	[ ]	ChIP-seq
[x]	[ ]	Flow cytometry
[x]	[ ]	MRI-based neuroimaging

Antibodies

Antibodies used	All anti-HIV antibodies used for this study were produced in the Kong lab or provided by Xueling Wu. Phosphatase-conjugated Goat anti-Human IgG used in ELISA was obtained from Southern Biotech.
Validation	All anti-HIV antibody constructs were validated by sequencing by Psomagen. Phosphatase-conjugated Goat anti-Human IgG was validated by the manufacturer for use in ELISA.

Eukaryotic cell lines

Policy information about cell lines

Cell line source(s)	FreeStyle 293-F cells were obtained from Invitrogen. ExpiCHO-S cells were obtained from Thermo Fisher Scientific.
Authentication	The authentication information of commercial cell lines is described on the manufacturer's website.
Mycoplasma contamination	All cell lines tested negative for mycoplasma contamination.
Commonly misidentified lines (See ICLAC register)	Cell lines used are not among the commonly misidentified lines.
